# Effect of alkyl chain length on the corrosion inhibition performance of 2-thioxo-2,3-dihydroquinazolin-4(1H)-one derivatives for carbon steel in HCl solution

**DOI:** 10.1038/s41598-026-40197-z

**Published:** 2026-03-31

**Authors:** Samir A. Abd El-Maksoud, W. Fathalla, Mohamed S. Saleh, Farid I. El-Dossoki

**Affiliations:** 1 Chemistry Department, Faculty of Science, Port Said University, Port Said, Egypt; 2 Chemistry Department, Faculty of Engineering, Suez Canal University, Ismailia, Egypt

**Keywords:** Quinazoline derivatives, Corrosion inhibition, Adsorption, Alkyl chain, Carbon steel, Chemistry, Materials science

## Abstract

This article investigates two quinazoline derivatives for reducing carbon steel corrosion in acidic media, commonly encountered in various chemical processes, which contribute significantly to corrosion. The structurally simple and easily synthesized compounds, 3-methyl-2-thioxo-2,3-dihydroquinazolin-4(1H)-one (Q-C1) and 3-butyl-2-thioxo-2,3-dihydroquinazolin-4 (1H)-one (Q-C4), were evaluated as corrosion inhibitors for carbon steel in 1.0 M HCl solution using chemical, electrochemical, surface analysis, and quantum computational methods, with particular attention to the effect of alkyl chain length on the inhibition efficiency. The inhibitory efficiency reached 88.91% for Q-C4 at 4.0 × 10^-5^, achieved during weight-loss testing on the carbon steel surface in 1.0 M HCl at 298 K. The temperature impact (ranging from 298 to 318 K) on the corrosion inhibition was investigated, revealing that with an increase in temperature, the inhibition efficiency diminishes. The kinetic and thermodynamic parameters were determined and analyzed to express the adsorption behavior of the studied inhibitors. The adsorption of the compounds on the carbon steel surface conforms to the Langmuir adsorption isotherm. The electrochemical measurements were conducted through the electrochemical impedance (EIS) and potentiodynamic polarization (PP) techniques, which confirm that the studied compounds were categorized as mixed-type inhibitors. EIS data reveal that charge-transfer resistance increases from 31.3 Ω cm^2^ in the uninhibited solution to 261.61 Ω cm^2^ in the inhibited solution of Q-C4. The surface of carbon steel was examined using scanning electron microscopy (SEM), energy dispersive X-ray (EDX), and Fourier-transform infrared spectroscopy (FT-IR), demonstrating the formation of a protective layer on the CS surface, mitigating the corrosion. Finally, the adsorption affinity of these compounds on the carbon steel surface was theoretically examined utilizing quantum calculations. The outcomes of the theoretical study in quantum chemistry were corroborated by data from chemical and electrochemical methods.

**Supplementary Information:**

The online version contains supplementary material available at 10.1038/s41598-026-40197-z.

## Introduction

Carbon steel is commonly employed as a building material in several sectors due to its excellent mechanical properties, affordability, and recyclability, particularly in the construction and concrete sectors^1^. Nonetheless, a significant problem related to the use of carbon steel alloys in these processes is their exposure to aggressive media, which can result in considerable metal corrosion^2,3^. This can be considered a drawback, as it might result in substantial industrial issues and threaten human safety^4^.

Corrosion is an inherent process that converts alloys and metals into more stable forms, such as oxides and sulfides, through direct contact with their surroundings. To address this issue, several approaches are available for corrosion mitigation, including cathodic protection, anodic protection, metallic and polymeric coatings, and modifying the corrosive environment through the use of corrosion inhibitors^5^. Industries and researchers frequently employ the latter technique. These compounds are added in small quantities to a corrosive medium, and must be environmentally safe, economically viable, and easy to use. Their presence hinders or entirely obstructs the dissolution reaction between the metal surface and its surroundings. Inhibitors may be classified as organic or inorganic substances that can adhere to the metal surface, blocking active corrosion sites, shielding the metal from the corrosive medium, and minimizing corrosion^6,7^.

Organic inhibitors are frequently employed to mitigate metal corrosion. Heterocyclic organic compounds with active sites such as sulfur, oxygen, and nitrogen in their structures show enhanced inhibitory efficiency^8,9^. Among these, nitrogen-containing substances have shown notable properties as efficient inhibitors for metals in HCl medium^10,11^. The inhibiting effect is generally attributed to the development of a physical and/or chemical adsorption protective barrier on the metal surface. Physisorption arises from electrostatic interaction between opposite charges in the metallic surface and inhibitor molecules, whereas chemisorption refers to the tendency of inhibitor molecules to share or transfer their charge to the metal surface to form a coordinated bond^12^. In fact, electron transfer is typically associated with transition metals that possess unoccupied electron orbitals. Heterocyclic compounds possess electron-donating atoms, nitrogen and oxygen, which may occupy the vacant orbitals of metals like iron with the necessary electrons, thus preventing their oxidation^13,14^. Moreover, the integration of alkyl chains into heterocyclic organic compounds has shown increased inhibitory efficacy. As the alkyl chain extends, the molecule’s hydrophobic nature increases, enabling greater surface coverage and offering further surface protection. Moreover, excessive chain length may induce steric hindrance, limiting the molecules’ proximity to the metallic surface and diminishing the effective contact area^15,16^.

Quinazoline derivatives are a promising class of organic inhibitors in mitigating corrosion rates since they are environmentally acceptable and safe to use in many applications^17–19^. Quinazoline compounds are aromatic heterocycles involving two nitrogen atoms in their ring, allowing their derivatives to interact with metallic surfaces^20^. Previous studies on quinazoline-based inhibitors have focused on structurally complicated, high-molecular-weight molecules, incorporating many substituents and difficult synthetic pathways^21–24^. Conversely, our research focuses on structurally simple, easily synthesized quinazoline compounds incorporating different alkyl substituents while still integrating essential heteroatoms (N, S, and O) in a configuration suitable for adsorption on the metal surface, thereby presenting economical and scalable options for industrial corrosion mitigation. The summarized purpose of this study was to evaluate the inhibitory effects of these compounds on steel in a 1.0 M HCl solution. The evaluation was conducted using weight loss and electrochemical methods at various inhibitor concentrations. Additionally, surface characterization techniques such as SEM–EDX and FTIR were employed to confirm the inhibition behavior.

## Experimental work

### Materials and chemicals

**Fig. 1:**
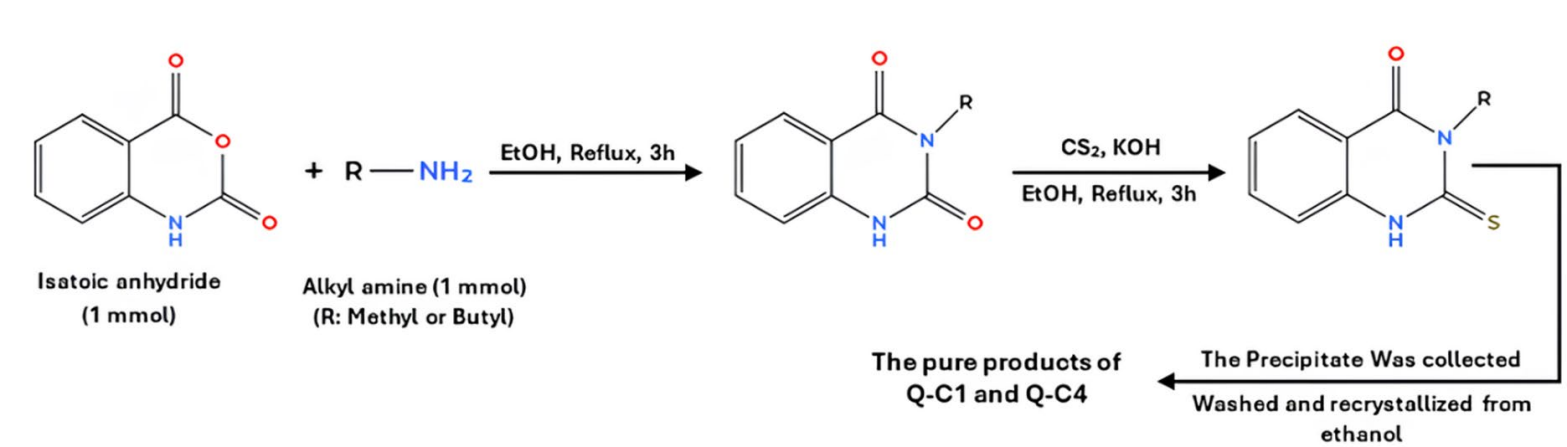
A schematic representation of the preparation of the studied quinazolinone derivatives.

Carbon steel specimens used for gravimetric and electrochemical measurements have the following chemical composition (wt%): 0.14% C, 0.1% Cr, 0.01% Ni, 0.024% Si, 0.5% Mn, 0.05% P, 0.05% S, and the balance Fe. The quinazolinone derivatives were synthesized following previously reported procedures^25^, as shown in Fig. 1. The molecular structures and analytical data of the synthesized inhibitors are presented in Table 1. A 5.0 M hydrochloric acid (HCl) stock solution was freshly prepared by diluting concentrated HCl (37%, Sigma-Aldrich) with double-distilled water. The solution was standardized using a sodium carbonate standard. A 1.0 M HCl solution was then prepared by diluting the stock solution and used as the blank corrosive medium. Stock solutions of the inhibitors (5 × 10^− 3^ M) were freshly prepared by dissolving the required amount of each compound in 10 mL of dimethylformamide (DMF), then diluting to 100 mL with absolute ethanol. Working solutions with concentrations ranging from 1 × 10^− 5^ to 4 × 10^− 5^ M were prepared by diluting the stock solution with double-distilled water to the desired concentration. When higher concentrations of quinazolinone derivatives (above 4.0 × 10^− 5^ M) were prepared, the resulting solutions were not fully clear. Consequently, all experiments were conducted using concentrations ≤ 4.0 × 10^− 5^ M.

**Table 1:**
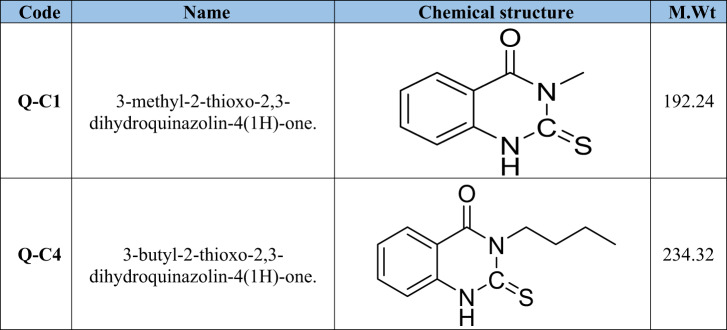
Nomenclature, chemical structures, and molecular weight of the studied compounds.

### Mass loss (ML) method

Carbon steel (CS) specimens with dimensions of 2.0 × 2.0 × 0.5 cm were polished using emery sheets of grades ranging from 400 to 1200, washed with double-distilled water, degreased with acetone, dried using filter papers, and accurately weighed. The samples were then immersed in 100 mL of 1.0 M HCl solution using glass hooks, both in the absence and presence of varying concentrations of Q-C1 and Q-C4 inhibitors, for 24 h at different temperatures (25–45 °C). Temperature control was maintained using a thermostatically regulated water bath with an accuracy of ± 0.1 °C. Samples were withdrawn at 4-hour intervals, rinsed, dried, and reweighed using a four-digit analytical balance. The corrosion rate (CR) and inhibition efficiency (IE) of the inhibitors were calculated accordingly. Each concentration was repeated three times, and the average value was reported.

### Electrochemical techniques

Electrochemical measurements were carried out using a CS Multichannel Potentiostat (Wuhan Corrtest Instruments Corp., Ltd., China) controlled via CS Studio software. A three-electrode glass cell setup was selected, consisting of a working electrode (carbon steel with a 1 cm² exposed area mounted on a copper rod and embedded in glass), a platinum sheet as the counter electrode, and an Ag/AgCl electrode as the reference.

The CS working electrodes were prepared in the same manner as for the ML tests. All measurements were conducted at 25 °C in 1.0 M HCl, with and without inhibitors. Before measurement, the working electrode was allowed to stabilize in solution until the open-circuit potential (OCP) was reached. Potentiodynamic polarization curves were recorded over a potential range of ± 100 mV relative to OCP at a scan rate of 0.5 mV/s. This scan window may not cause surface changes^26,27^. Electrochemical parameters were obtained by extrapolating Tafel regions to analyze the inhibition performance.

**Fig. 2:**
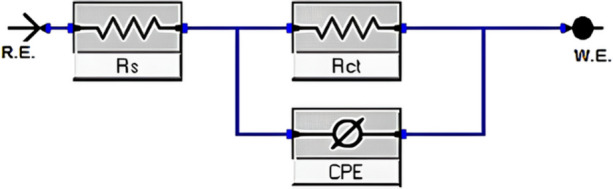
The equivalent circuit used for fitting EIS graphs.

Electrochemical impedance spectroscopy (EIS) measurements were performed at OCP with a 10 mV AC perturbation over the frequency range of 100 kHz to 0.1 Hz, using 10 points per frequency decade. The resulting Nyquist and Bode plots were analyzed using an appropriate equivalent circuit model to extract the charge transfer resistance (R_ct_) and double-layer capacitance (C_dl_). The R_ct_ values were used to estimate corrosion rates and inhibitor efficiency. The analysis of the experimental data was conducted by aligning the results to the appropriate equivalent circuit presented in Fig. 2.

### Surface characterization

The surface morphology and elemental composition of uninhibited and inhibited CS samples were examined using scanning electron microscopy (SEM, JEOL JSM-6510LV, Japan) coupled with energy-dispersive X-ray spectroscopy (EDX). For this purpose, the CS samples were immersed in 1.0 M HCl with and without the highest concentration of Q-C1 for 24 h at 25 °C and 45 °C. After immersion, the samples were dried at ambient temperature before analysis.

### Fourier transform infrared spectroscopy (FTIR)

Fourier transform infrared (FTIR) spectroscopy was employed to investigate the surface chemistry of CS after immersion. Spectra were recorded using a Bruker FTIR spectrometer in the mid-IR range (500–4000 cm⁻¹) with a spectral resolution of 4 cm⁻¹ and 128 scans. Analyses were evaluated on the CS surfaces after 24-hour submersion in 1.0 M HCl at 25 °C and 45 °C with the optimum inhibitor concentration, and the results were compared to the FTIR spectra of the pure solid inhibitor.

### DFT study

Density Functional Theory (DFT) is a prevalent computational approach to compare the chemical reactivity of a series of compounds. In the present study, DFT calculations were conducted via the Gaussian 09 software, with input files generated via GaussView. Initially, the molecular geometries were optimized using the semi-empirical PM6 method to obtain appropriate starting structures. Subsequently, the geometries were further refined and analyzed using the B3LYP functional within the framework of DFT. The calculations employed the high-level 6-311 + + G (d, p) basis set to guarantee precise and reliable results.

The electronic characteristics of the investigated compounds, including the highest occupied molecular orbital energy (${E_{HOMO}}$) and the lowest unoccupied molecular orbital (${E_{LUMO}}$) were evaluated, and various DFT variables such as ionization potential ($IE$), energy band gap (ΔE), electronic potential ($\mu$), global hardness ($\eta$), electrophilicity ($\omega$), the fraction of electrons transferred (${ΔN}$), electron affinity ($EA$), electronegativity ($\chi$), and chemical softness (σ) were calculated using these equations:

$$\Delta E={E_{LUMO}} - ~{E_{HOMO}}$$

$$IE=~ - ~{E_{HOMO}}$$

$$EA= - ~{E_{LUMO}}$$

$$\mu =~\frac{{{E_{LUMO}}+~{E_{HOMO}}}}{2}$$

$$\chi =~\frac{{IE+~EA}}{2}$$

$$\eta =~\frac{{IE - ~EA}}{2}~$$

$$\omega =~\frac{{{\mu ^2}}}{{2~\eta }}$$

$${ΔN}=\frac{{{\chi _{Fe}} - ~{\chi _{inh.}}}}{{2\left( {{\eta _{Fe}}+~{\eta _{inh.}}} \right)}}$$

$$\sigma =~1/\eta$$

A theoretical value for the work function (${\chi _{Fe}}$= 4.82 eV) of the Fe (1 1 0) plane, and a global hardness of ${\eta _{Fe}}$= 0 were used.

## Results and discussion

### Mass loss measurements

The corrosion behavior of CS in 1.0 M HCl was examined both in the absence and presence of various inhibitor concentrations. The obtained data, including corrosion rates (${C_R}$) and inhibition efficiency (%IE) for each concentration are presented in Table 2. Equation (1) is used to evaluate the inhibition efficiency and the surface coverage (θ) of the investigated inhibitor compounds^23,28^. The mass loss study was conducted under different conditions, including various inhibitor concentrations, immersion durations, and temperatures.

1 $$\begin{array}{*{20}c} {\% IE~ = ~\theta ~ \times 100~ = \left( {1 - ~\frac{W}{{W^{^\circ } }}} \right) \times 100~} \\ \end{array}$$

where (W°) and (W) represent the average weight loss without as well as with the existence of the inhibitor, respectively.

#### Effect of concentration

**Fig. 3:**
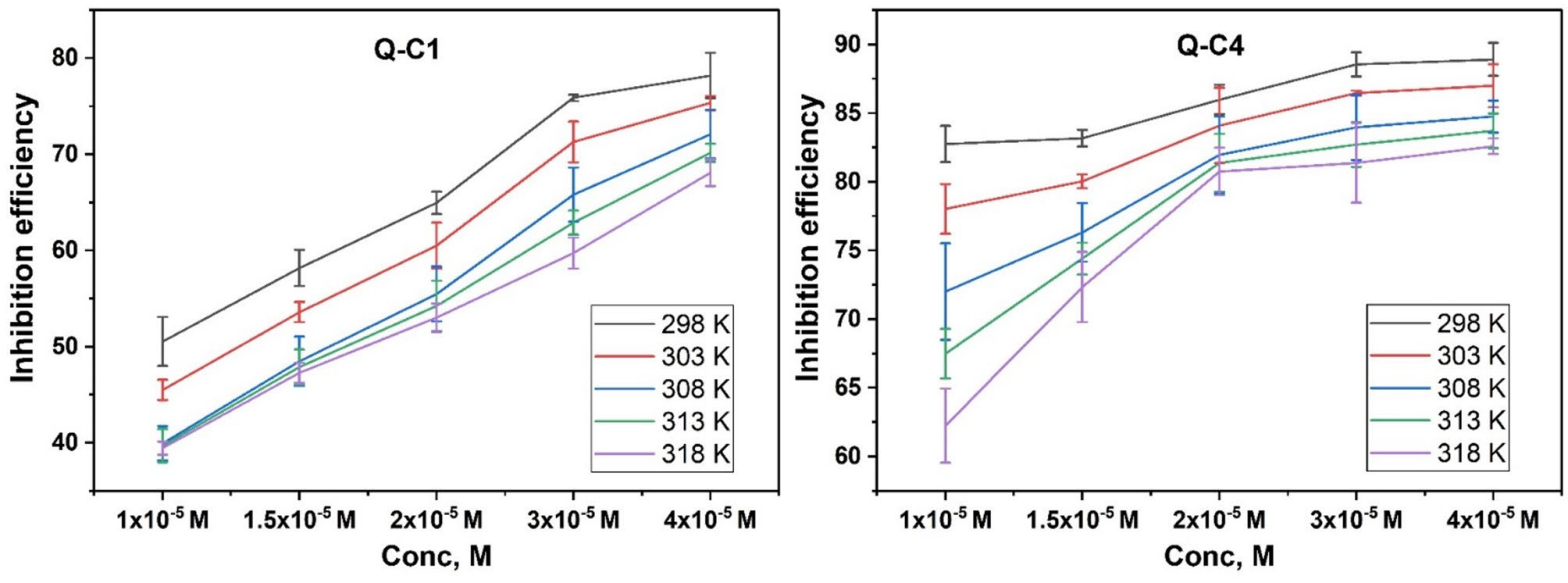
Inhibition efficiency with various concentrations of Q-C1 and Q-C4 at different temperatures.

Table 2 shows that the corrosion rates are markedly reduced with the addition of organic inhibitors. Moreover, the efficiency of inhibition increases with increasing concentrations of the inhibitors, as shown in Fig. 3. This behavior was attributed to the protective barrier formed on the carbon steel surface^29,30^. It was also found that the inhibitor Q-C4 exhibited a greater inhibitory efficiency. The adsorption of the inhibitor molecules onto the CS surface is mediated by the presence of benzene rings and single (C–N) bonds, which block active corrosion sites effectively. The adsorption process is further enhanced by transferring charge among the delocalized π-electrons in the benzene ring and the empty d-orbitals in the iron atoms^31,32^. Furthermore, the inclusion of heteroatoms, such as sulphur and nitrogen, in the synthesized molecules’ structures improves their adsorption ability, making them efficient corrosion inhibitors^33,34^. Inhibitor Q-C4 may demonstrate enhanced adsorption onto the metal surface due to increased -CH_2_ groups attached to the aromatic ring, resulting in improved isolation of the metal-medium interaction^35^.

**Table 2:** Corrosion rates of CS after 16 h in 1.0 M HCl at various temperatures, 298–318 K, and with different concentrations of inhibitors.

Inh.	Conc (M)	298 K		303 K		308 K		313 K		318 K	
		CR (mg cm^− 2^ min^− 1^)	%IE	CR (mg cm^− 2^ min^− 1^)	%IE	CR (mg cm^− 2^ min^− 1^)	%IE	CR (mg cm^− 2^ min^− 1^)	%IE	CR (mg cm^− 2^ min^− 1^)	%IE
Blank	1.0 M HCl	0.0066	–	0.0094	–	0.0133	–	0.0199	–	0.0297	–
Q-C1	1.0 × 10^− 5^	0.0033	50.53	0.0051	45.49	0.0080	39.93	0.0120	39.69	0.0180	39.44
	1.5 × 10^− 5^	0.0028	58.18	0.0044	53.58	0.0069	48.47	0.0104	47.88	0.0157	47.28
	2.0 × 10^− 5^	0.0023	64.96	0.0037	60.50	0.0059	55.47	0.0091	54.25	0.0140	53.00
	3.0 × 10^− 5^	0.0016	75.88	0.0027	71.28	0.0046	65.80	0.0074	62.89	0.0120	59.73
	4.0 × 10^− 5^	0.0014	78.20	0.0023	75.34	0.0037	72.10	0.0059	70.16	0.0095	68.08
Q-C4	1.0 × 10^− 5^	0.0011	82.76	0.0021	78.03	0.0037	72.01	0.0065	67.49	0.0112	62.25
	1.5 × 10^− 5^	0.0011	83.17	0.0019	80.04	0.0032	76.32	0.0051	74.41	0.0082	72.34
	2.0 × 10^− 5^	0.0009	85.98	0.0015	84.10	0.0024	81.97	0.0037	81.37	0.0057	80.75
	3.0 × 10^− 5^	0.0008	88.55	0.0013	86.45	0.0021	83.97	0.0034	82.72	0.0055	81.38
	4.0 × 10^− 5^	0.0007	88.91	0.0012	87.00	0.0020	84.76	0.0032	83.71	0.0052	82.60

#### Effect of immersion time

**Fig. 4:**
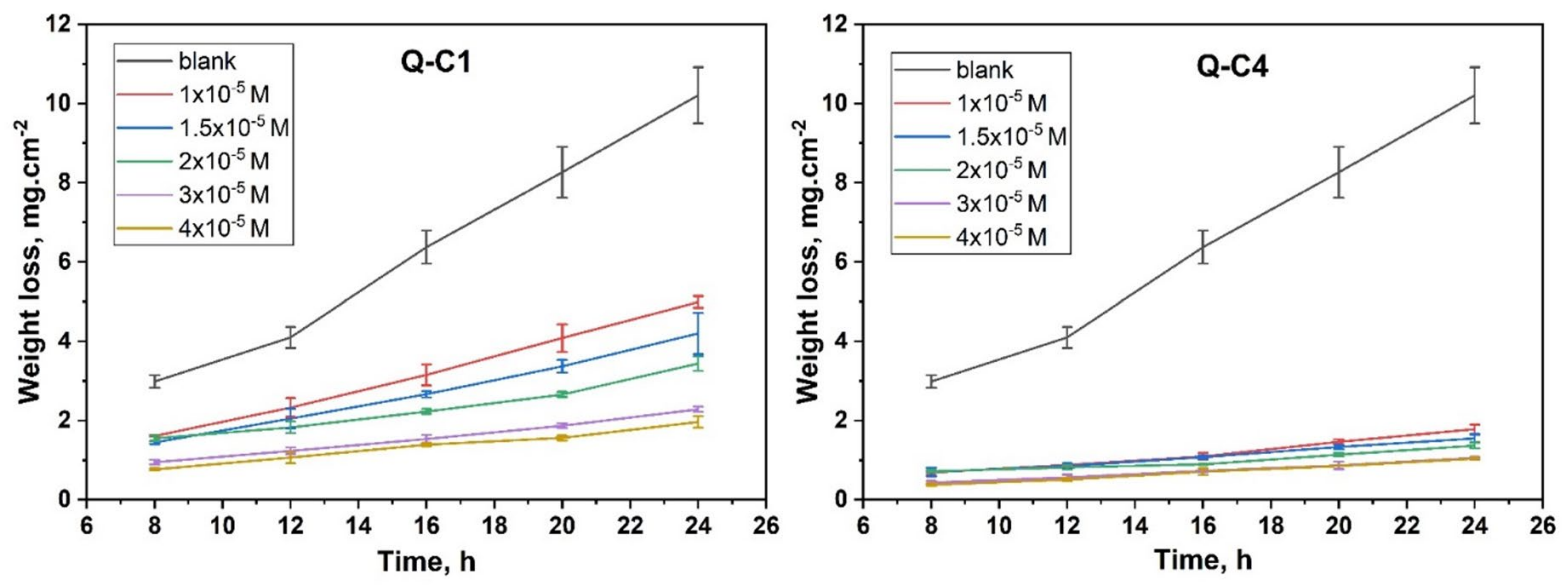
WL-Time graphs for CS in 1.0 M HCl without and with various concentrations of Q-C1 and Q-C4 at 298 K.

To investigate the stability behavior of quinazoline inhibitors, the influence of immersion time was examined for all concentrations, as shown in Fig. 4. The graph indicates that the maximum inhibitory efficacy was seen at the optimal concentration (4 × 10^− 5^ M), the efficiency was gradually increased with the increase in immersion period until a specific period (16 h) was reached and then became stabilized. The formation of an outer barrier on the CS surface may elucidate this observation, which protects the metal during a prolonged duration of testing^36,37^.

#### Effect of temperature

The durability of the protective layer on the metal substrate can be determined by studying the impact at various temperatures at each inhibitor concentration^38^. The dissolution of CS in 1.0 M HCl was studied over the temperature range of 298 to 318 K, with and without the addition of inhibitors. Table 2 indicates that the efficiency of inhibition diminishes with rising temperature, and an identical pattern was observed across all concentrations examined. This may result from the removal of adsorbed inhibitor molecules from the metal’s outer layer when the temperature rises^39,40^.

**Activation thermodynamic parameters** were studied using Arrhenius and transition state Eqs. (2) and (3)^41^.

2 $$\log {C_R}=\left( {\frac{{ - ~E_{a}^{*}}}{{2.303RT}}} \right)+\log A~~$$

3 $$\begin{array}{*{20}{c}} {\log \left( {\frac{{{C_R}}}{T}} \right)=\left( {log\frac{{RT}}{{Nh}}} \right)+\left( {\frac{{\Delta {S^*}}}{{2.303R}}} \right)+\left( {\frac{{ - ~\Delta {H^*}}}{{2.303RT}}} \right)~~} \end{array}$$

where ${C_R}$ indicates the corrosion rate resulting from ML measurements, R indicates the gas constant, and T indicates the absolute temperature, $E_{a}^{*}~$donates the apparent activation energy, A indicates the Arrhenius frequency factor, N indicates Avogadro’s number, $\Delta {H^*}$ and $\Delta {S^*}$ refers to the enthalpy and entropy of activation, whereby h refers to Planck’s constant.

**Fig. 5:**
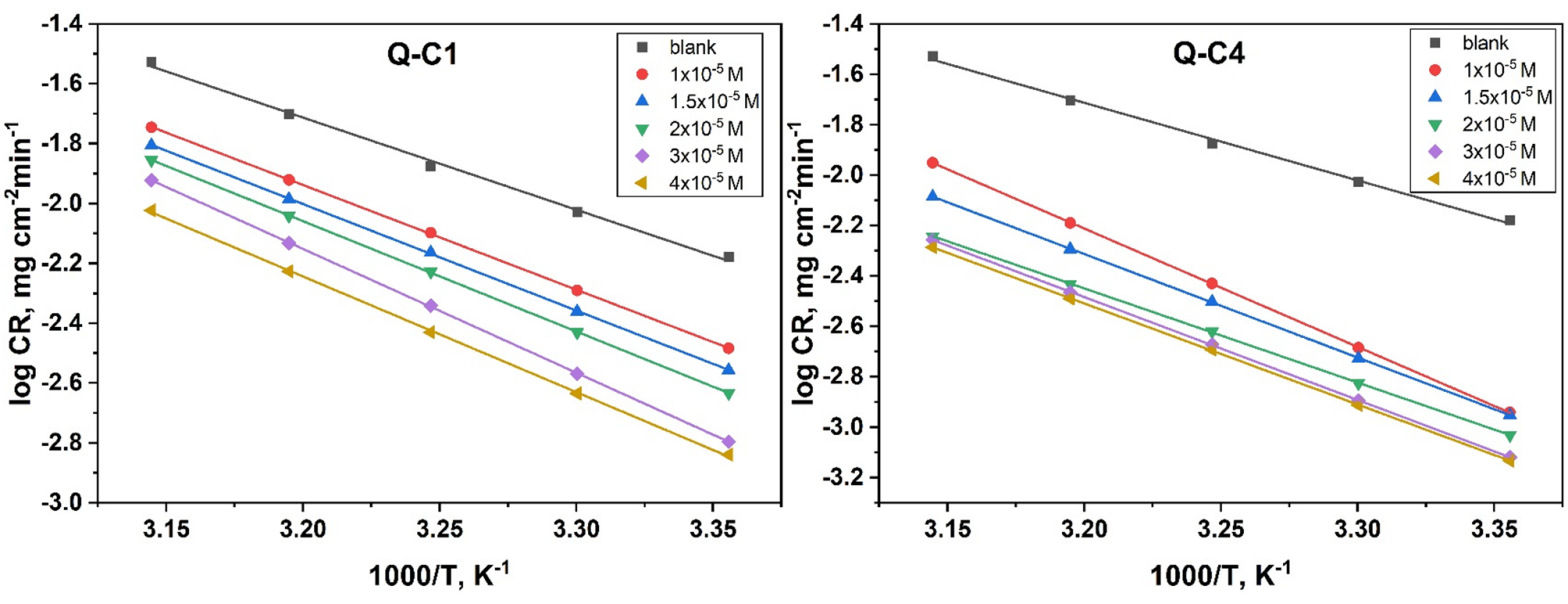
Arrhenius graphs for the corrosion of CS in 1.0 M HCl without as well as after adding different doses of Q-C1 and Q-C4.

Figure 5 illustrates Arrhenius plots of (log ${C_R}$) vs. (1000/T) for the corrosion of CS in 1.0 M HCl of various concentrations of inhibitors at different temperatures (298–318 K). Straight lines with a slope of $\left( {\frac{{ - ~E_{a}^{*}}}{{2.303R}}} \right)$ and an intercept (log A) were estimated^42^. Table 3 shows that the values of $E_{a}^{*}$ in the presence of quinazolinone derivatives are greater than that in their absence, indicating a physisorption mechanism^43^. Nevertheless, this explanation cannot be considered definitive owing to competition with adsorbed water, whose removal from the surface needs an amount of activation energy. Other parameters can be used to evaluate the dominance of chemisorption or physisorption, arising from other adsorption experimental data^44^.

**Fig. 6:**
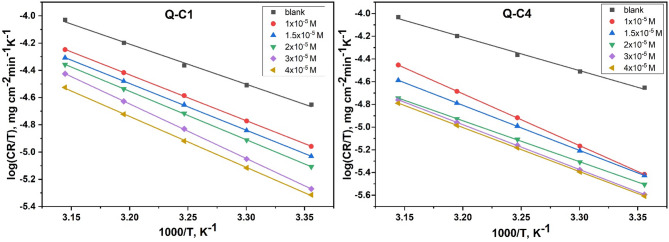
Transition state graphs for the corrosion of CS in 1.0 M HCl without as well as after adding different doses of Q-C1 and Q-C4.

The transition state plots of (log ${C_R}$/T) vs. (1000/T) for Q-C1 and Q-C4 are illustrated in Fig. 6. Straight lines were obtained with slopes equal to $\left( {\frac{{ - ~\Delta {H^*}}}{{2.303RT}}} \right)$ and intercepts equal to $\left( {log\frac{{RT}}{{Nh}}} \right)+\left( {\frac{{\Delta {S^*}}}{{2.303R}}} \right)$, which are utilized for the calculation of $\Delta {H^*}$ and $\Delta {S^*}$ as shown in Table 3. The positive values of $\Delta {H^*}$ indicate that CS dissolves endothermically. The enthalpy values obtained in the existence of two compounds are higher than that of the free solution, indicating that the corrosion reaction proceeds spontaneously^45^. In addition, the negative $\Delta {S^*}$ values for inhibited solutions are less negative than that for the uninhibited solution. This may result from the desorption of numerous water molecules that were previously adsorbed on the surface of the metal and the adsorption of less disordered, bigger inhibitor molecules on the carbon steel surface, and the competition between them contributes to increasing disorder of the system^46,47^.

**Table 3:** The activation parameters for the corrosion of CS in 1.0 M HCl, without as well as after adding different concentrations of Q-C1 and Q-C4.

Comp.	Conc. (M)	Activation parameters		
		$E_{a}^{*}$ (kJ. mol^− 1^)	$\Delta {H^*}$ (kJ. mol^− 1^)	-$\Delta {S^*}$ (J mol^− 1^ K^− 1^)
Q-C1	Blank	58.98	56.42	97.60
	1.0 × 10^− 5^	67.01	64.45	76.21
	1.5 × 10^− 5^	68.18	65.62	73.70
	2.0 × 10^− 5^	70.61	68.05	67.00
	3.0 × 10^− 5^	79.24	76.68	41.17
	4.0 × 10^− 5^	74.03	71.47	59.59
Q-C4	1.0 × 10^− 5^	89.94	87.38	8.09
	1.5 × 10^− 5^	78.62	76.06	46.24
	2.0 × 10^− 5^	71.56	69.00	71.45
	3.0 × 10^− 5^	78.20	75.64	50.85
	4.0 × 10^− 5^	76.80	74.24	55.81

**Fig. 7:**
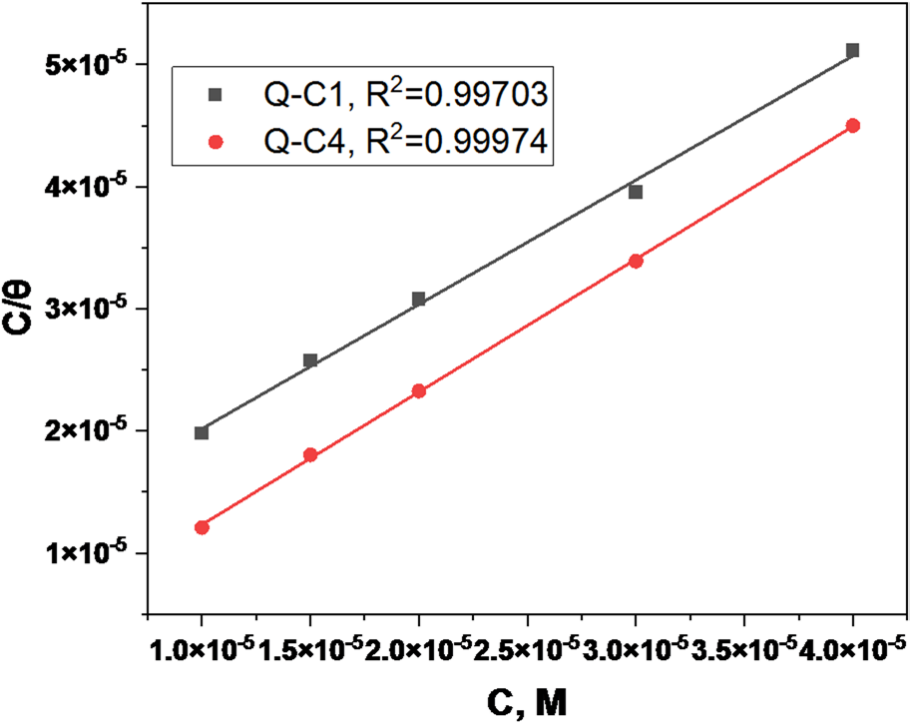
Langmuir adsorption isotherm of Q-C1 and Q-C4 for the corrosion of CS at 298 K.

The interaction between inhibitor molecules and active sites on the CS surface can identify the kind of adsorption. The adsorption process may be influenced by several variables, such as the kind of metal, the chemical structure of the molecules under investigation, and the properties of the acids involved^48^. To elucidate the nature of adsorption, many isotherms were evaluated; however, the Langmuir isotherm yields the optimal straight-line fit, suggesting the formation of a mono-adsorbed layer^49^. The plots of ${C_{inh}}/\theta$ against of ${C_{inh}}$ provide a linear relationship, Fig. 7, having a regression coefficient (R^2^) exceeding 0.99, indicating that the adsorption of quinazolinone derivatives on the CS surface conforms to the Langmuir adsorption isotherm presented by (Eq. 4)^50^:

4 $$\begin{array}{*{20}{c}} {{C_{inh}}/\theta =~1/{K_{ads}}+{C_{inh}}~} \end{array}$$

Where ${K_{ads}}$ represents the equilibrium constant of the adsorption process, and its value was determined from the intercept of the Langmuir plot. ${K_{ads}}~$is employed to determine the standard Gibbs energy of adsorption $\Delta G_{{ads}}^{^\circ }$ by the next (Eq. 5):

5 $$\begin{array}{*{20}{c}} {\Delta G_{{ads}}^{^\circ }=~ - 2.303~RTlog\left( {{K_{ads}} \times 55.5} \right)~} \end{array}$$

In the above equation, R represents the universal gas constant, T is the thermodynamic temperature in kelvin, and the number 55.55 indicates the concentration of water in solution in mol/L. The obtained values of ${K_{ads}}$ and $\Delta G_{{ads}}^{^\circ }$ for each organic inhibitor are listed in Table 4. The greater ${K_{ads}}$values suggest the higher stability of the adsorbed layer on CS, and it was observed that these values decrease with increasing temperature. This indicates that lower temperatures are beneficial for the inhibitory process. Moreover, inhibitor Q-C4 with high values of ${K_{ads}}$ implies enhanced adsorption of this inhibitor and therefore enhanced corrosion inhibition efficacy^51,52^. Whereas, the values of $\Delta G_{{ads}}^{^\circ }$were employed to elucidate the adsorption type of inhibitors. These values are negative, as indicated in Table 4, signifying that the adsorption of inhibitor molecules onto the surface of CS is spontaneous. Many studies indicate that the adsorption of inhibitors is classified as physisorption if the $\Delta G_{{ads}}^{^\circ }$values are close to − 20 kJ mol^− 1^ or less negative, and chemisorption when they are around − 40 kJ mol^− 1^ or more negative. The $\Delta G_{{ads}}^{^\circ }$ values for Q-C1 and Q-C4 range from − 38.76 to − 43.94 kJ.mol^− 1^, indicating that the inhibitor adsorption action on the CS surface may be a combination of chemisorption and physisorption^53^.

**Fig. 8:**
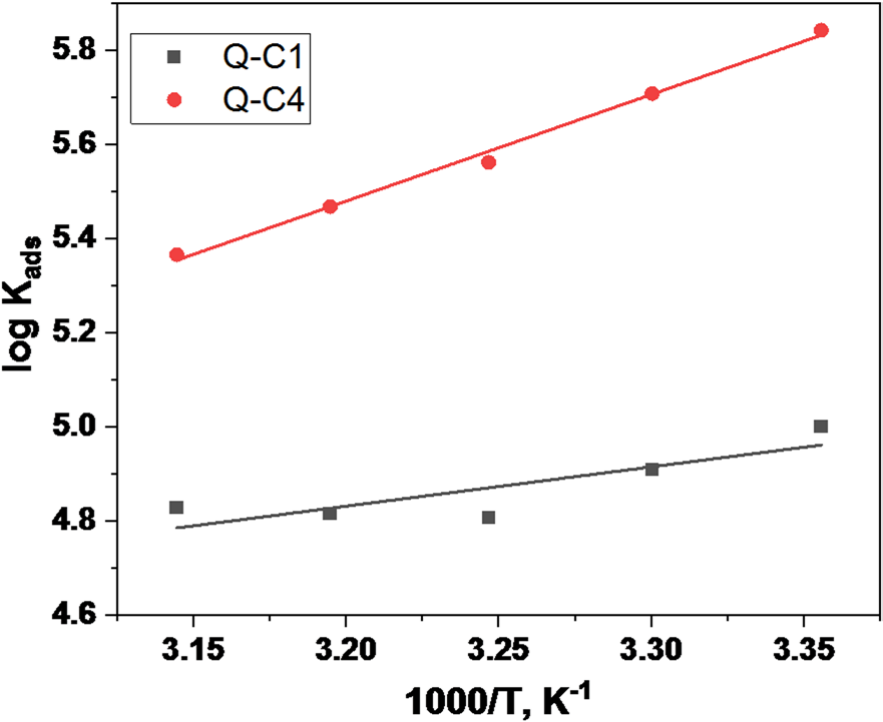
A log K_ads_ versus (1/T) plots of Q-C1 and Q-C4.

Equation 6 (Van’t Hoff equation) may be used to figure out the heat of adsorption ($\Delta H_{{ads}}^{^\circ }$) by plotting log ${K_{ads}}$ versus 1/T as shown in (Fig. 8).

6 $$\begin{array}{*{20}{c}} {\log {K_{ads}}=~\frac{{ - \Delta H_{{ads}}^{^\circ }}}{{2.303RT}}+constant~} \end{array}$$

The standard adsorption entropy ($\Delta S_{{ads}}^{^\circ }$) can be estimated from (Eq. 7).

7 $$\begin{array}{*{20}{c}} {\Delta G_{{ads}}^{^\circ }=~\Delta H_{{ads}}^{^\circ } - ~T\Delta S_{{ads}}^{^\circ }~} \end{array}$$

As shown in Table 4, the $\Delta H_{{ads}}^{^\circ }$ values are negative, demonstrating the exothermic nature of the adsorption process, which means releasing heat; consequently, raising the temperature will promote the desorption of inhibitor molecules. Furthermore, the entropy $\Delta S_{{ads}}^{^\circ }~$of the solvent diminishes in the presence of Q-C4 than Q-C1. This indicates that the replacement of water molecules with inhibitor molecules in the solution leads to the formation of a more organized adsorption layer at the metal/solution contact^54–56^.

**Table 4:** Adsorption isotherm parameters for Q-C1 and Q-C4 on the metal surface of CS at various temperatures.

Inh.	Temp. (°K)	$\:{K}_{ads}$ $\:\times\:{10}^{4}$(M^− 1^)	$\:-\:\varDelta\:{G}_{ads}^{^\circ\:}$ (kJ. mol^− 1^)	$\:{-\:\varDelta\:H}_{ads}^{^\circ\:}$ (kJ. mol^− 1^)	$\:\varDelta\:{S}_{ads}^{^\circ\:}$ (J. mol^− 1^ K^− 1^)
Q-C1	298	10.00	38.48	16.00	75.44
	303	8.12	38.60		74.59
	308	6.42	38.64		73.49
	313	6.55	39.32		74.50
	318	6.74	40.02		75.53
Q-C4	298	69.73	43.29	43.38	-0.29
	303	51.11	43.24		-0.47
	308	36.54	43.09		-0.94
	313	29.41	43.23		-0.50
	318	23.26	43.30		-0.27

### Electrochemical measurements

#### Open circuit potential (OCP)

**Fig. 9:**
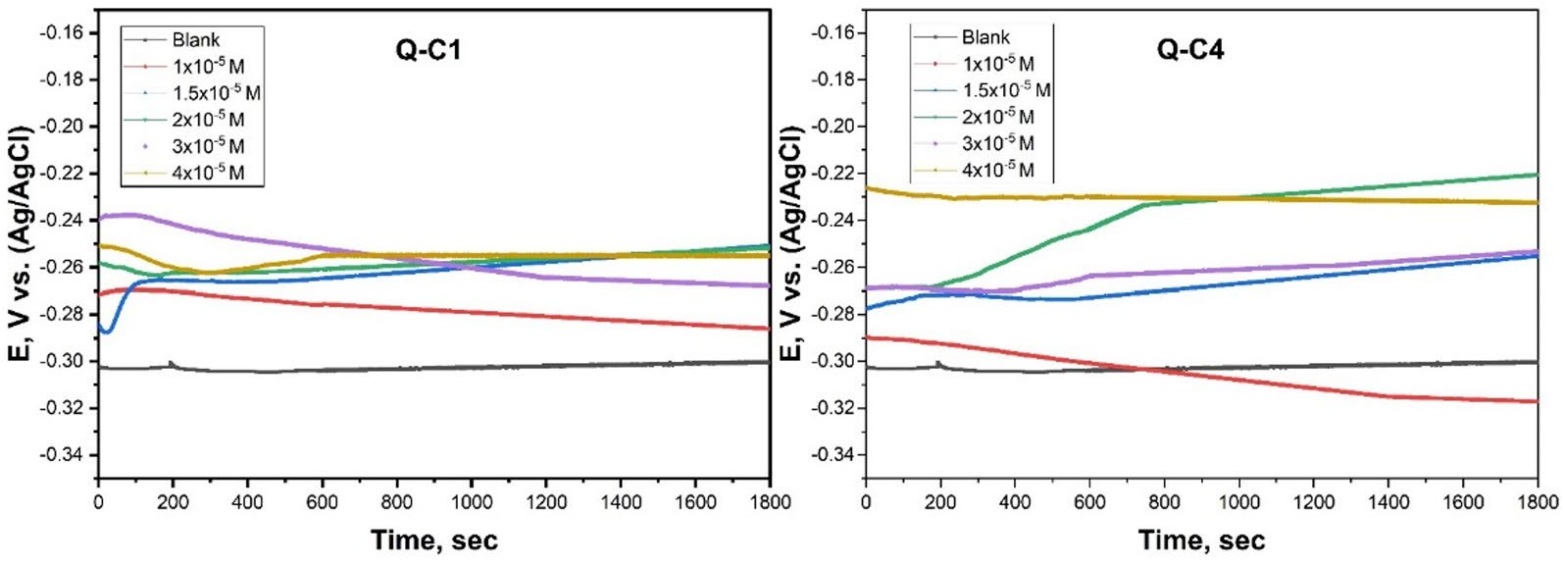
OCP of carbon steel with and without adding various concentrations of Q-C1 and Q-C4 at 298 K.

A stable OCP must be established before conducting electrochemical studies. The working electrode must be allowed to stabilize in a condition where the voltage remains relatively constant throughout time. This ensures that the measurements obtained during PDP and EIS are accurate and reflect the system’s true electrochemical behavior. Following 10 min of each concentration test, the OCP curves reached a linear pattern. Figure 9 illustrates the OCP versus time curves (in seconds). The presence of Q-C1 and Q-C4 resulted in the OCP curves stabilizing at more positive values relative to the blank, signifying the development of a protective layer on the CS surface^57^.

#### Potentiodynamic polarization measurements

**Fig. 10:**
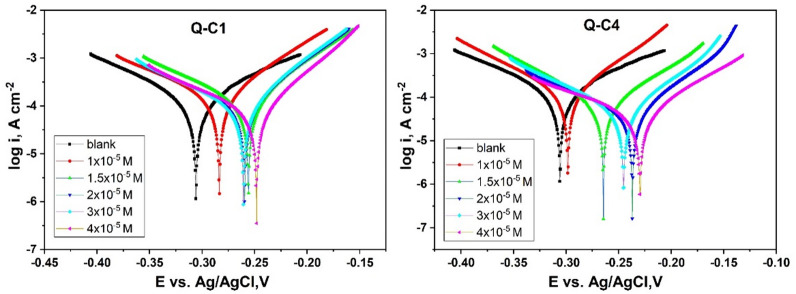
PP curves for the corrosion of CS in 1.0 M HCl without as well as after adding various concentrations of Q-C1 and Q-C4 at 298 K.

Figure 10 illustrates polarization curves for CS in 1.0 M HCl before as well as after the addition of various concentrations of Q-C1 and Q-C4 at 298 K. The extrapolation of the anodic and cathodic curves provides the current densities (i_corr_) and corrosion potentials (E_corr_) at the intersection point. These extracted electrochemical parameters, as well as Tafel slopes (cathodic (${\beta _c}$) and anodic (${\beta _a}$)) are displayed in Table 5. The i_corr_ values were employed to calculate the inhibition efficiency, %IE, via the next equation (Eq. 8):

8 $$\begin{array}{*{20}{c}} {\% I{E_{PP}}~=~\theta ~ \times 100~=\left( {1 - ~\frac{{{i_{corr}}}}{{i_{{corr}}^{^\circ }}}} \right)~ \times 100~} \end{array}$$

Where ${i_{corr}}$ and $i_{{corr}}^{^\circ }$ denote the corrosion current densities for CS electrode in the HCl media with and without the use of the inhibitor, respectively.

The investigation of the Table (5) revealed an obvious reduction in the ${i_{corr}}$ values for solutions comprising Q-C1 and Q-C4 inhibitors with respect to the uninhibited solution and an increase in %IE. It is also noticed that the Tafel slopes changed slightly, suggesting that the synthesized quinazolinone derivatives did not influence the corrosion inhibition mechanism^58,59^. This can be clarified by the fact that the quinazolinone unit (the polar moiety) within the Q-C1 and Q-C4 molecules is coupled directly to the CS surface through unsaturated π-electrons and the lone pair electrons located in S and N atoms. Conversely, the carbon chain (the non-polar moiety) orients vertically and/or horizontally to the CS surface, repelling corrosive species in the corrosive medium and forming a barrier against chemical attacks^60,61^.

The literature indicates that if the variation between uninhibited and inhibited systems in the E_corr_ values exceeds 85 mV, the inhibitor can be categorized as anodic or cathodic. In contrast, for a mixed-type inhibitor, the change in value is equal to or less than 85 mV. In our case, the maximum shift is 82 mV, suggesting that Q-C1 and Q-C4 inhibitors can be considered mixed-type inhibitors, influencing the anodic as well as cathodic reactions^62–64^. However, in the inhibited solutions, E_corr_ values shifted along a less negative path parallel to the blank, indicating that these inhibitors are more effective on the anodic reaction (mild steel dissolution) rather than on the cathodic reaction (H^+^ ion reduction). Consequently, it is reasonable to claim that these compounds function as mixed-type inhibitors with a primary anodic inhibitive influence^65,66^. Both the cathodic (βc) and anodic (βa) Tafel slopes in inhibited solutions exhibited a similar shift of approximately 30 mV when compared to the uninhibited solution. This behavior of anodic and cathodic processes also confirms the mixed-type nature of the inhibitor. The Tafel polarization tests yield the same order of inhibitory efficiencies for the two compounds as deduced from mass loss measurements. The PDP findings of the examined quinazoline derivatives may be compared to other quinazoline compounds that have been reported as corrosion inhibitors, as shown in Table 6.

**Table 5:** Corrosion parameters derived from the extrapolation of CS Tafel curves at 298 K.

Inh.	Conc, (M)	${i_{corr}},$ µA cm^− 2^	-E_corr_, mV (vs. Ag/AgCl)	${\beta _a}$ mV dec^− 1^	${\beta _c}$ mV dec^− 1^	C.*R*, Mpy	$\theta$	$\% I{E_{PP}}$
Q-C1	Blank	318$\:\pm\:$0.38	311.02	90.50	129.6	146.71	….	….
	1.0 × 10^− 5^	183$\:\pm\:$0.24	283.62	74.27	131.8	84.43	0.4245	42.45
	1.5 × 10^− 5^	154$\:\pm\:$0.09	255.9	68.70	144.5	71.05	0.5157	51.57
	2.0 × 10^− 5^	131$\:\pm\:$0.19	259.53	66.96	158.9	60.44	0.5881	58.81
	3.0 × 10^− 5^	126$\:\pm\:$0.34	260.57	65.81	153.0	58.13	0.6038	60.38
	4.0 × 10^− 5^	94$\:\pm\:$0.43	248.13	59.32	141.9	43.37	0.7044	70.44
Q-C4	1.0 × 10^− 5^	147$\:\pm\:0.19$	298.29	67.90	97.7	67.82	0.5377	53.77
	1.5 × 10^− 5^	84$\:\pm\:$0.45	264.5	82.30	92.9	38.75	0.7358	73.58
	2.0 × 10^− 5^	59$\:\pm\:$0.56	236.92	67.20	147.7	27.22	0.8145	81.45
	3.0 × 10^− 5^	54$\:\pm\:$0.77	245.32	63.01	99.5	24.91	0.8302	83.02
	4.0 × 10^− 5^	52$\:\pm\:$0.76	229.61	74.86	158.7	23.99	0.8365	83.65

**Table 6:** Comparison between the corrosion Inhibition effectiveness of the examined inhibitors and other established Quinazoline derivatives for steel in 1.0 M HCl by potentiodynamic polarization.

No.	Inhibitor	Concentration	IE%	References
1	3-methyl-2-thioxo- 2,3-dihydroquinazolin-4(1H)-one	4.0 × 10^− 5^ M	70.44	Present study
2	3-butyl-2-thioxo-2,3- dihydroquinazolin-4(1H)-one.	4.0 × 10^− 5^ M	83.65	
3	2-(quinolin-2-yl) quinazolin-4(3H)-one	7.4 × 10^− 5^ M	81.20	^67^
4	3-propyl-2-thioxo- 2,3-dihydroquinazolin-4(1H)-one (Q-Inh)	4.0 × 10^− 5^ M	81.76	^68^
5	3-methyl-2-(4-nitrophenyl) quinazolin-4(3H) one (QZ-NO_2_)	1.0 × 10^− 5^ M	82.20	^23^
6	3-methyl-2-phenylquinazolin- 4(3H)-one (QZ-H)	1.0 × 10^− 5^ M	81.55	
7	2,3-dihydroquinazolin- 4(1H)-ones (ZB3)	1.0 × 10^− 4^ M	84.02	^69^
8	2,3-dihydroquinazolin- 4(1H)-ones (ZB4)	1.0 × 10^− 4^ M	82.60	

#### EIS measurements

**Fig. 11:**
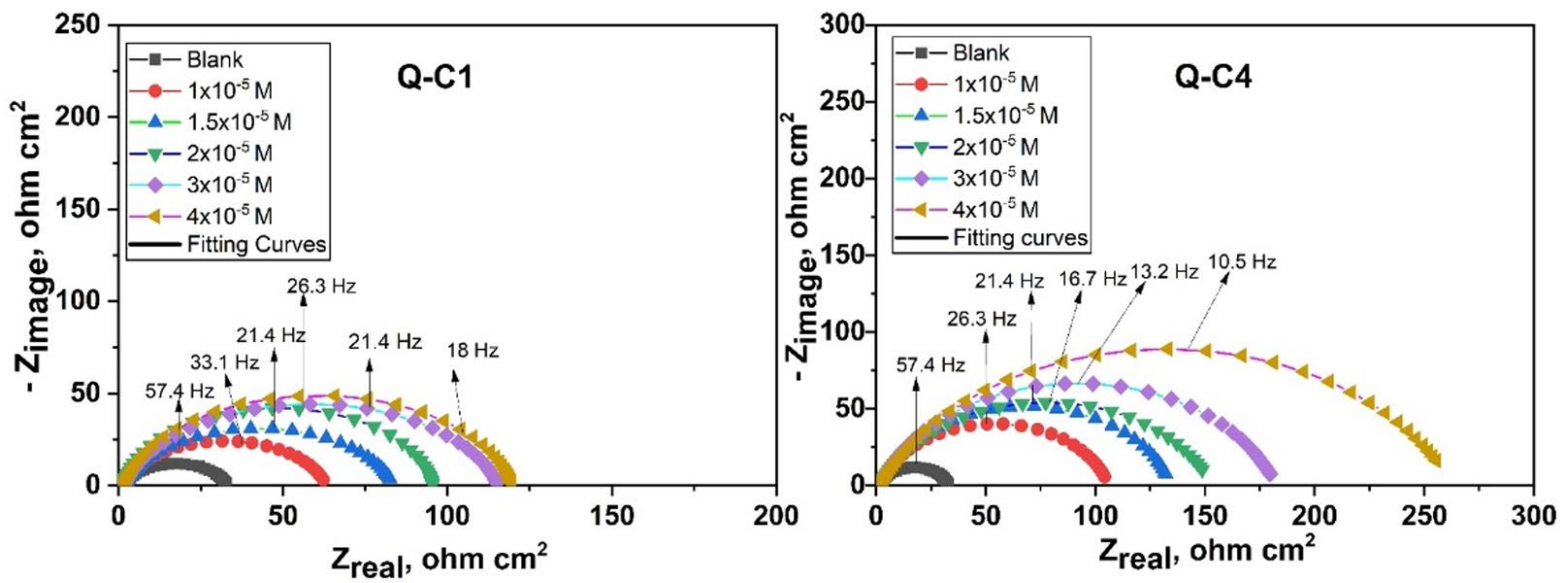
Nyquist for CS in 1.0 M HCl in the absence and existence of different concentrations of Q-C1 and Q-C4 at 298 K.

EIS was conducted to provide a deep understanding of the protective mechanism of organic inhibitors, yielding valuable insights into the system interface^70^. Nyquist plots (Fig. 11) display one capacitive loop, showing that the corrosion process was mainly charge transfer, and the diameter of the semicircles was enhanced with increasing inhibitor concentration. This result implies that an increase in the concentration of inhibitor molecules on the substrate correlates with an increase in its impedance. It is observed that these semicircle widths for Q-C1 < Q-C4 reveal that a longer chain length enhances inhibitory effectiveness^71^.

**Fig. 12:**
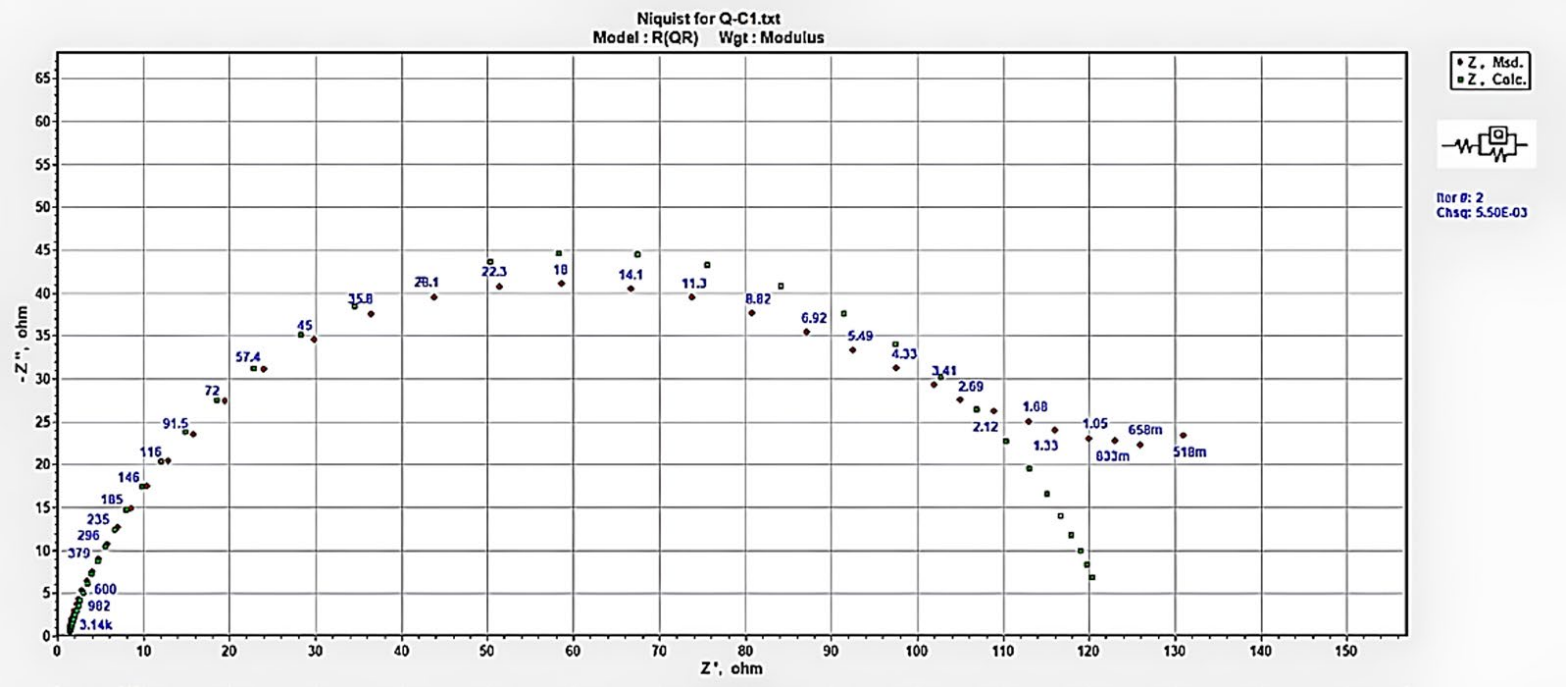
The experimental data were fitted to an equivalent circuit via the ZSimp software.

The imperfect circular form of the capacitive loops results from frequency dispersion caused by surface roughness, the presence of porous layers, dislocations, and surface inhomogeneities^72,73^. Therefore, the experimental data analysis was performed by correlating the findings with the equivalent circuit shown in Fig. 1, and the measured data was assessed by fitting to an equivalent circuit via the ZSimp software Fig. 12^74^. This circuit consists of a solution resistance (R_s_) in series with a charge transfer resistance (${R_{ct}}$) that is parallel with a constant phase element (CPE) that replaces a pure double-layer capacitor (C_dl_) to offer a more precise fit, reflecting surface heterogeneity^75,76^. The CPE (n) parameter reflects the inhomogeneity or roughness of the electrode surface. The n values for inhibited solutions do not show a specific trend in increasing or decreasing compared to the n values obtained for the uninhibited solution, suggesting that the formed film of inhibitor molecules may represent both adsorption modes, comprising compact chemically adsorbed regions alongside more loosely bound physically adsorbed regions^77,78^.

**Fig. 13:**
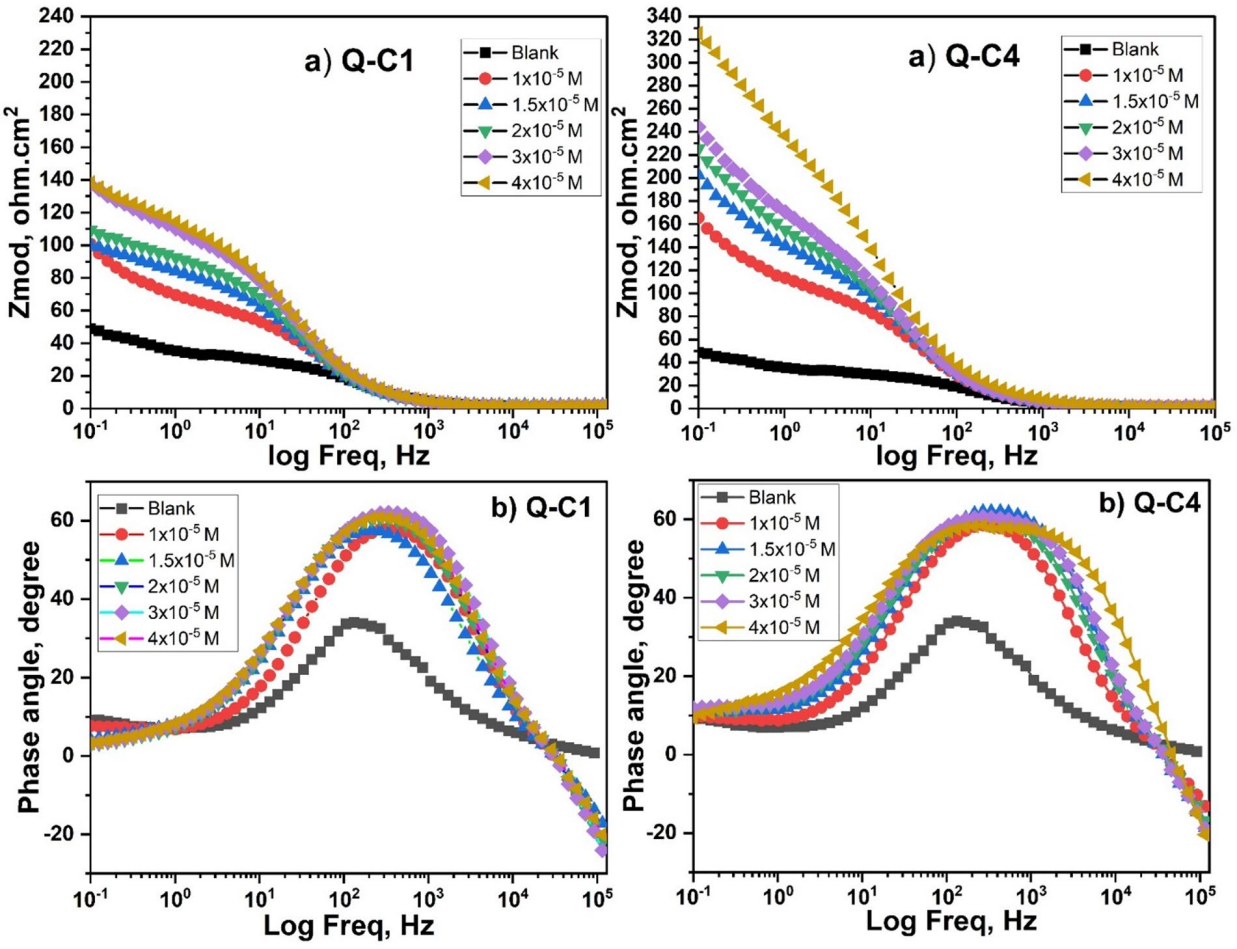
(a) Plots of Bode modulus and (b) plots of Bode phase for CS in 1.0 M HCl in the absence and existence of different concentrations of Q-C1 and Q-C4 at 298 K.

Figure 13 illustrates the Bode plots for the two inhibitors, revealing that at low frequencies, a greater impedance modulus is noted with increasing inhibitor concentrations, maintaining the same curve shape. This results in enhanced protection without altering the corrosion mechanism (charge transfer), and Q-C4 provides more effective protection for CS than Q-C1 due to its higher impedance. The phase angle values in Bode plots for inhibited specimens are significantly greater than those of the uninhibited specimen, suggesting that the surface becomes notably smoother due to the creation of a barrier of protection by inhibitor molecules on the CS surface. The broadening of the single maximum in the Bode plots further supports the protective film formation by inhibitor molecules^79,80^. Table 7 describes the impedance variables derived from the experimental data fitting. The values of ${R_{ct}}$ increase as the inhibitor concentration rises, resulting in enhanced inhibition efficiency (%IE) that can be calculated according to Eq. (9).

9 $$\begin{array}{*{20}{c}} {\% I{E_{EIS}}~=~\theta ~ \times 100~=\left( {1 - ~\frac{{R_{{ct}}^{^\circ }}}{{{R_{ct}}}}} \right) \times 100~} \end{array}~$$

where ${R_{ct}}$ and $R_{{ct}}^{^\circ }$ reflects the charge transfer resistances for CS electrode in the HCl media with and without the inhibitor addition, respectively.

While ${C_{dl}}~$values fall. The following Eq. (10) was employed to calculate the$~{C_{dl}}$ at the maximum frequency (${f_{max}}$).

10 $${C_{dl}}~=~1~/~2~\pi ~{f_{max}}~{R_{ct}}~$$

The values of ${C_{dl}}$ generally correspond to the Helmholtz formula, which is determined by the product of air permittivity, local dielectric constant, and surface exposed, with all terms being divided by the thickness of the developed layer^81,82^. Therefore, the significant decline in ${C_{dl}}~$results obtained for the inhibited solution, in contrast to the uninhibited one, is ascribed to the displacement of water molecules from the metal surface, which are substituted for organic molecules at the steel/solution interface by adsorption. This fact significantly reduces the local dielectric constant while simultaneously increasing the film thickness due to the size of Q-C1 and Q-C4 in comparison to water molecules, leading to diminished double-layer capacitance^83^.

EIS measurements demonstrate enhanced protection of the metallic surface due to the adsorption of inhibitor molecules, which impede the corrosive process, and align with results attained using the PP and ML methods.

**Table 7:** EIS parameters of CS in 1.0 M HCl and in the presence of various doses of Q-C1 and Q-C4 at 298 K.

Inh.	Conc, (M)	${R_S}$, (${Ω .cm^2}$)	${R_{ct}}$, (${Ω .cm^2}$)	*n*	Y₀$\times$10^− 6^, ($\mu {{\mathrm{Ω}}^{ - 1}}~{s^n}c{m^{ - 2}})$	${C_{dl,}}$ $\left( {\mu F~c{m^{ - 2}}} \right)$	Goodness of fit	$\theta$	$\% I{E_{EIS}}$
Blank	1.0 M HCl	1.568	31.32$\:\pm\:$0.49	0.823	198.3	88.58	0.0068	–	–
Q-C1	1.0 × 10^− 5^	1.422	62.96$\:\pm\:$0.69	0.826	207.5	76.41	0.0015	0.5026	50.26
	1.5 × 10^− 5^	1.746	82.49$\:\pm\:$0.64	0.807	260.3	73.40	0.0090	0.6203	62.03
	2.0 × 10^− 5^	1.305	96.26$\:\pm\:$0.89	0.809	243.7	77.30	0.0064	0.6747	67.47
	3.0 × 10^− 5^	8.260	118.55$\:\pm\:$1.4	0.776	266.7	62.77	0.0037	0.7358	73.58
	4.0 × 10^− 5^	1.167	121.30$\:\pm\:$1.8	0.807	197.5	72.93	0.0055	0.7418	74.18
Q-C4	1.0 × 10^− 5^	2.077	103.96$\:\pm\:$1.2	0.826	157.6	58.24	0.0014	0.6988	69.88
	1.5 × 10^− 5^	1.463	132.14$\:\pm\:$2.5	0.826	153.6	56.31	0.0029	0.7630	76.30
	2.0 × 10^− 5^	1.508	151.24$\:\pm\:$1.9	0.790	190.2	63.05	0.0014	0.7929	79.29
	3.0 × 10^− 5^	0.935	179.96$\:\pm\:$2.5	0.757	232.3	67.03	0.0026	0.8260	82.60
	4.0 × 10^− 5^	0.857	261.61$\:\pm\:$2.7	0.718	241.1	57.97	0.0039	0.8803	88.03

### Surface characterization

#### Scanning electron microscopy

**Fig. 14:**
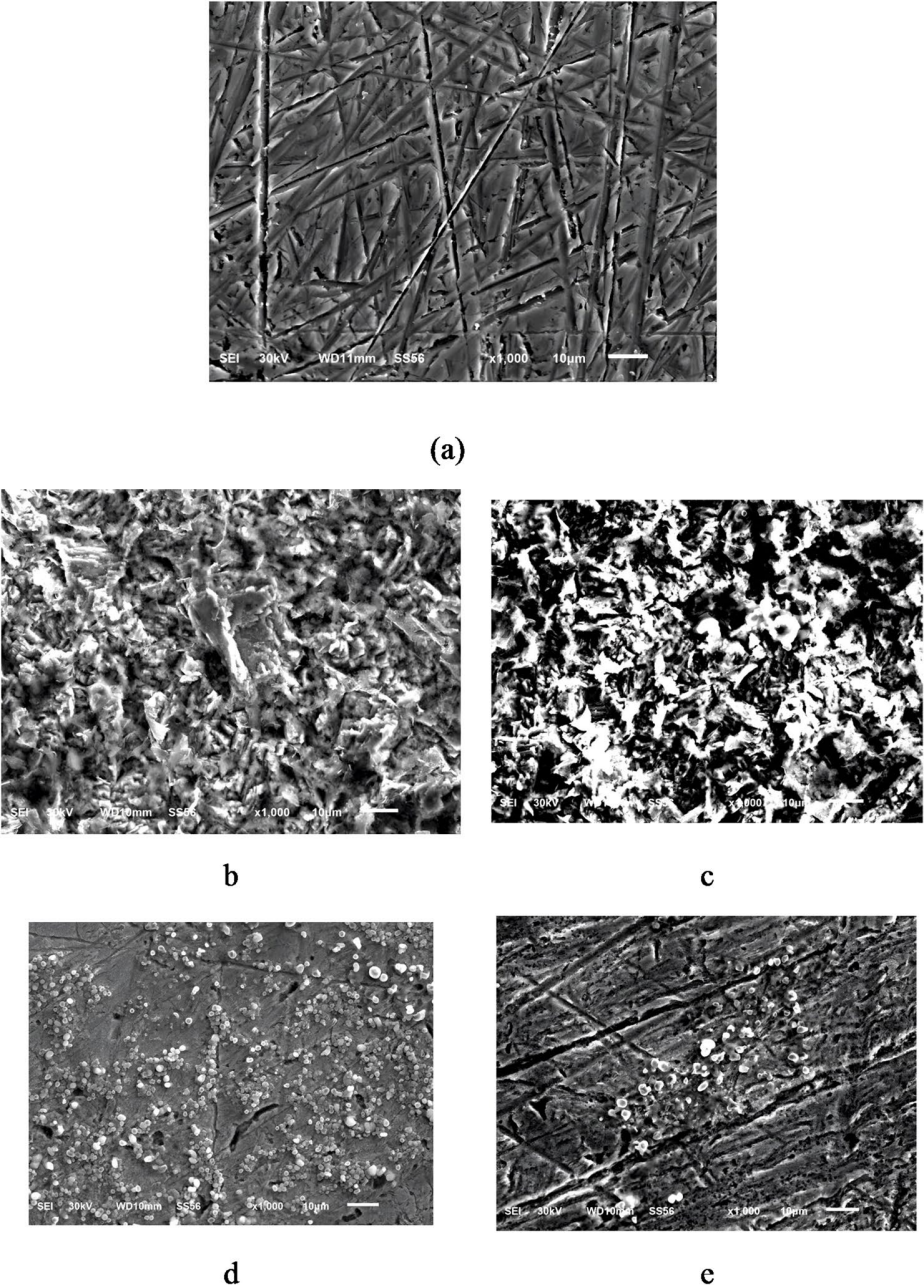
SEM images of (a) Free C-steel, (b) blank C-steel at 298 K, (c) blank C-steel at 318 K, (d) 4 × 10^− 5^ M (Q-C1) C-steel at 298 K, (e) 4 × 10^− 5^ M (Q-C1) C-steel at 318 K.

SEM micrographs (Fig. 14) of the surfaces of CS were obtained to examine the alteration that happened during the corrosion process in both the absence and presence of inhibitors at 298 K and 318 K. Figure 14a depicts the abraded surface of the CS image prior to immersion in a 1.0 M HCl solution; the abrasion scratches are clearly seen on the CS surface. Figures 14b-c illustrate the CS sample immersed in uninhibited 1.0 M HCl for 24 h, showing significant corrosion characterized by cracks and pits resulting from a rapid corrosion attack. It can be believed that the CS surface experienced considerable damage in the absence of inhibitors^84,85^. Conversely, in the presence of 4 × 10^− 5^ M of Q-C1 (Figs. 14d-e), a relatively smoother and less corroded topography of the CS surface is clear, indicating the development of a protective film on the surface of CS. Furthermore, it is evident from (Fig. 14e) that surface coverage diminishes with rising temperatures owing to the desorption of organic inhibitors at elevated temperatures^86^.

#### EDX studies

**Fig. 15:**
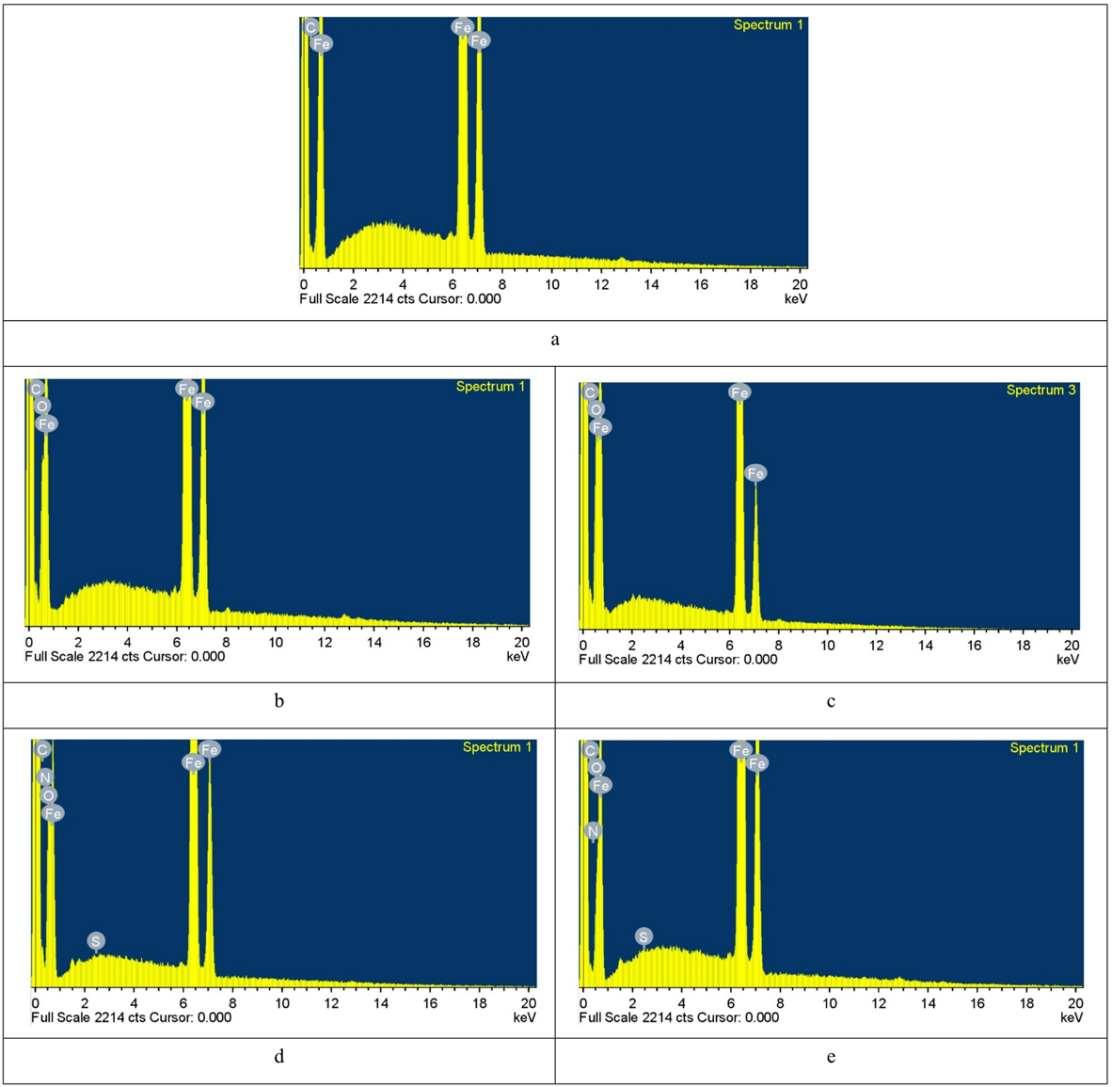
EDX spectra of CS (a) Free C-steel, (b) blank C-steel at 298 K, (c) blank C-steel at 318 K, (d) 4 × 10^− 5^ M (Q-C1) C-steel at 298 K, (e) 4 × 10^− 5^ M (Q-C1) C-steel at 318 K.

The data in EDX spectra at 298 K and 318 K are shown in (Fig. 15a-e). Figure 15a shows the EDX spectrum of the abraded steel surface, revealing the characteristic peaks of the components comprising the carbon steel sample. Figures 15b–c show the EDX spectrum of the uninhibited CS sample with an extra oxygen peak, perhaps related to the formed iron oxides on the steel surface as corrosion products^87^. Inhibited samples of Q-C1 at maximum concentration (Fig. 15d, e) show a spectrum with extra peaks characteristic of the existence of N, S, and an increase in the intensity of the O peak, indicating the formation of an adsorbed layer of inhibitor on carbon steel. Table 8 shows the percentage atomic compositions of the identified elements across all samples, revealing that Fe peaks are diminished in the presence of the inhibitor, particularly at 298 K. Additionally, at elevated temperatures, the intensity of the N atom signal decreased, confirming the adsorbed film stability on CS at lower temperatures^88^.

**Table 8:** Surface characteristics (wt%) of CS.

Samples	Weight%				
	Fe	O	C	*N*	S
C-steel	92.50	0.0	7.50	0.0	0.0
1.0 M HCl at 298 K	68.51	11.26	20.23	0.0	0.0
1.0 M HCl at 318 K	72.30	12.10	15.60	0.0	0.0
Q-C1 at 298 K	35.51	25.41	22.26	16.82	0.01
Q-C1 at 318 K	63.58	10.45	17.02	8.94	0.02

### Fourier‑transform infrared spectroscopy (FT‑IR) analysis

**Fig. 16:**
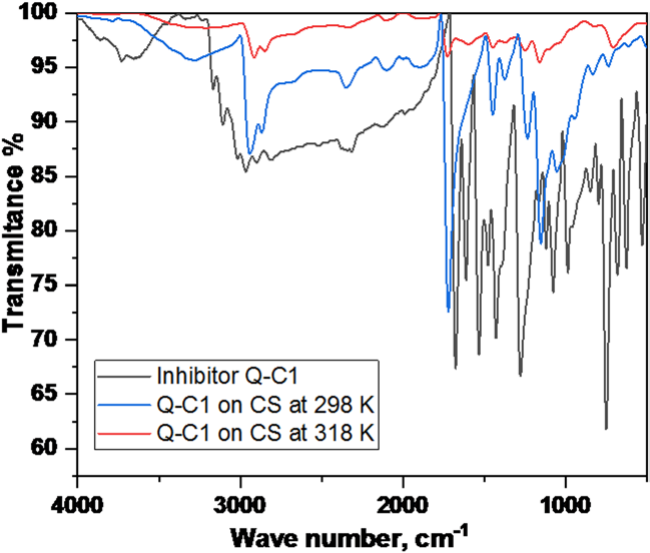
FTIR spectra for compound Q-C1 at 298 K and 318 K.

FT-IR spectrum analysis gives an in-depth investigation of the chemical reactions occurring at the interface between the corrosive medium and the carbon steel surface. Figure 16 compares the IR patterns of the pure solid inhibitor Q-C1 and the inhibitor adsorbed at various temperatures on the steel surface. The IR spectra of the analyzed samples reveal that the characteristic peaks of the inhibited carbon steel coupons are similar to those of the pure inhibitor^89^.

The IR spectrum of the examined pure inhibitor exhibits recognizable peaks at 3164, 3012, 1671, 1611, and 1276 cm^− 1^, indicative of N$\:-$H, C$\:-$H, C = O, C = C, and C$\:-$N stretching vibration frequencies, respectively, with an additional peak seen in the low-frequency zone at 750 cm^− 1^corresponding to C-S. The spectra of the inhibitor applied to the CS surface exhibit positional variations in IR absorption peaks, such as a noticeable shift in N$\:-$H and C = O bands to greater wavenumber, indicating a reduction in bond length and an enhancement in bond strength due to decreased electron density on these heteroatoms after their contact with the metal surface, suggesting that the inhibitor molecules are effectively adsorbed onto the steel surface^90^.

At 298 K, the spectrum shows low transmittance, indicating increased adsorption of infrared radiation, suggesting a larger concentration of inhibitor molecules adsorbed on the metal surface. Conversely, desorption of inhibitor molecules occurs at 318 K, resulting in high transmittance.

### DFT study

**Fig. 17:**
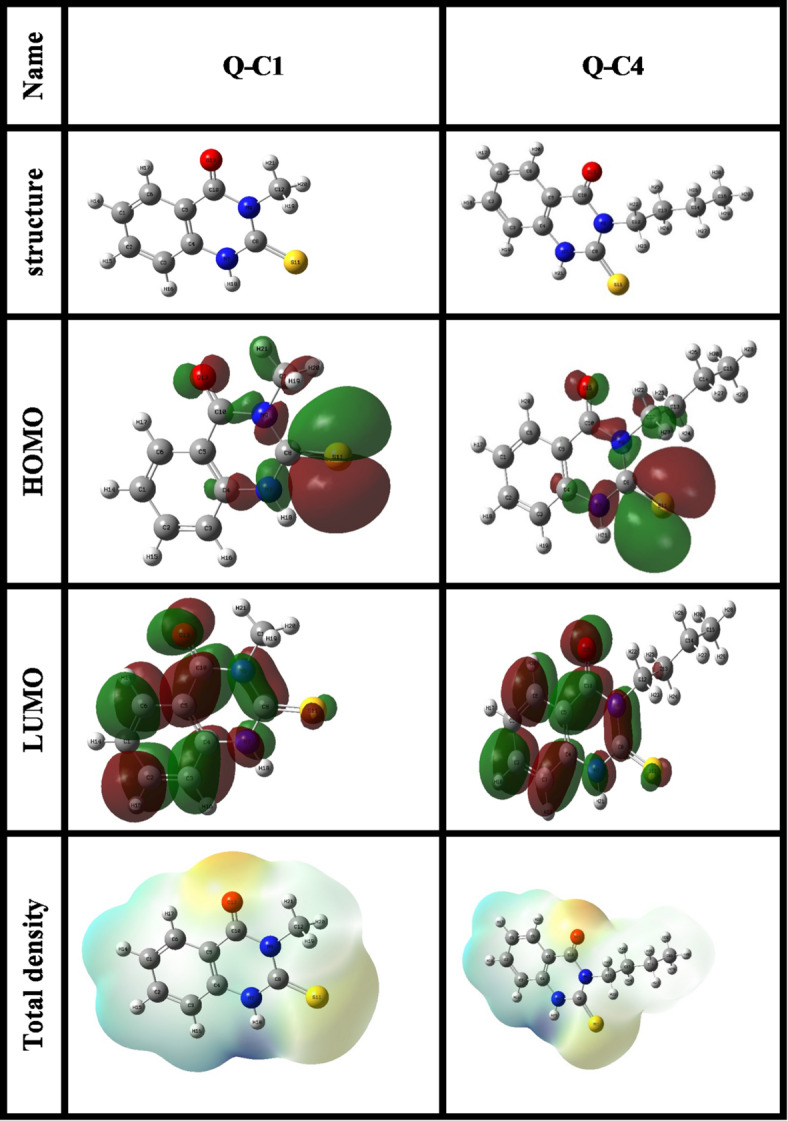
Optimized structures, HOMO, LUMO electronics density, and ESP distributions for Q-C1 and Q-C4. All molecular structures were generated using Gaussian 09 W 9.5 Revision D.01 (Gaussian, Inc., Wallingford, CT, USA; https://gaussian.com/).

The molecular reactivity can be investigated by density functional theory (DFT). The optimized structures of the studied compounds are shown in Fig. 17. These structures have the most stable ground state and planar geometries, whereby the arrangement of atoms facilitates optimal interactions with the metallic surface. The co-planarity of the N- and O-heteroatoms and p-electron systems in the molecular structures contributes to their effective corrosion inhibition efficiency^91^.

The electronic distribution of frontier molecular orbitals significantly affects the reactivity and corrosion resistance of the molecules. Figure 17 displays the HOMO diagrams that show areas with a greater tendency to donate electrons to electron-deficient entities such as metal vacant orbitals, while the LUMO diagrams reveal the parts of molecules that tend to receive electrons from filled metal orbitals. To reach the best compound’s capability to donate and accept electrons, a higher ${E_{HOMO}}$ value and lower ${E_{LUMO}}$ value is required^92,93^. Furthermore, the difference between ${E_{HOMO}}$ and ${E_{LUMO}}$, denoted as ΔE. A smaller ΔE value indicates a greater inhibitory effect. Table 8 displays that the compound Q-C4 has a relatively higher ${E_{HOMO}}$ value and lower ΔE value, which confirms the higher reactivity and inhibition efficiency for Q-C4 when compared to Q-C1.

According to the Hard and Soft Acids and Bases theory, two interacting species, i.e., a metallic surface and an inhibitory molecule, should exhibit matching hardness (η) and softness (σ) values. Consequently, the interaction strength enhances as the softness (σ) increases and the hardness (η) decreases. Thus, the compound Q-C4 with a higher σ value and a smaller η value becomes slightly softer and has a stronger propensity to interact with the metallic surface when compared to Q-C1^94^.

Moreover, the lowest $\chi$ value for Q-C4 indicates a higher tendency to easily release its loosely bound electrons compared to Q-C1. This facilitates its interaction with the metal and improves the adsorption and inhibitory efficacy^95^. The trend of ∆N implies that Q-C4 can donate a greater fraction of electrons to the metal than Q-C1, and the incorporation of a butyl chain in Q-C4 demonstrates a stronger donation power by sharing its lone pair, due to its slightly greater + I effect compared to a methyl chain in Q-C1. The trend in the quantum chemical parameter values in Table 9 aligns with the previously reported experimental results of inhibition efficiency.

The principle of atomic charge serves as an effective technique for studying molecular features. The Mulliken population study is used to evaluate atomic charges across the atoms in chemical compounds. The Mulliken distribution of charge for the inhibitors is shown in Table 10. Heteroatoms such as sulfur (S), oxygen (O), and nitrogen (N) demonstrate the greatest charge density levels, making them the most reactive sites for electrophilic interaction. This interaction promotes the development of a protective layer on carbon steel, effectively hindering further corrosion in acidic solutions^96^.

**Table 9:** Computational variables calculated for Q-C1 and Q-C4.

Quantum parameters	Q-C1	Q-C4
${E_{HOMO}}$, eV	-0.2288	-0.2271
${E_{LUMO}}$, eV	-0.0763	-0.0752
∆E, eV	0.1525	0.1520
$IE$, eV	0.2288	0.2271
$EA$, eV	0.0763	0.0752
$\mu$, eV	-0.1525	-0.1511
$\chi$, eV	0.1525	0.1511
$\eta$, eV	0.0762	0.0760
$\sigma$, eV^− 1^	13.1191	13.1614
∆N, eV	30.6062	30.7363
$\omega$, eV	0.1526	0.1504

**Table 10:** Mulliken charges on individual atoms for Q-C1 and Q-C4.

	Q-C1	Q-C4
Mulliken charges	1 C 0.125757	1 C -0.387197
	2 C -0.167871	2 C 0.013635
	3 C -0.221898	3 C -0.160286
	4 C -1.303039	4 C 0.258545
	5 C 1.336695	5 C 0.270161
	6 C -0.201394	6 C -0.243938
	7 N -0.005908	7 N -0.003372
	8 C -0.283572	8 C -0.305231
	9 N 0.087345	9 N 0.210646
	10 C 0.042209	10 C -0.127671
	11 S 0.071801	11 S -0.017197
	12 C -0.211458	12 C -0.333745
	13 O -0.351695	13 C 0.089104
	14 H 0.077334	14 C -0.163795
	15 H 0.080269	15 O -0.327393
	16 H 0.072439	16 C -0.561863
	17 H 0.106591	17 H 0.065837
	18 H 0.265376	18 H 0.078810
	19 H 0.162714	19 H 0.064096
	20 H 0.162704	20 H 0.127602
	21 H 0.155601	21 H 0.285299
		22 H 0.187641
		23 H 0.179927
		24 H 0.130865
		25 H 0.133147
		26 H 0.088083
		27 H 0.097423
		28 H 0.122592
		29 H 0.116729
		30 H 0.111545

## Mode of inhibition

**Fig. 18:**
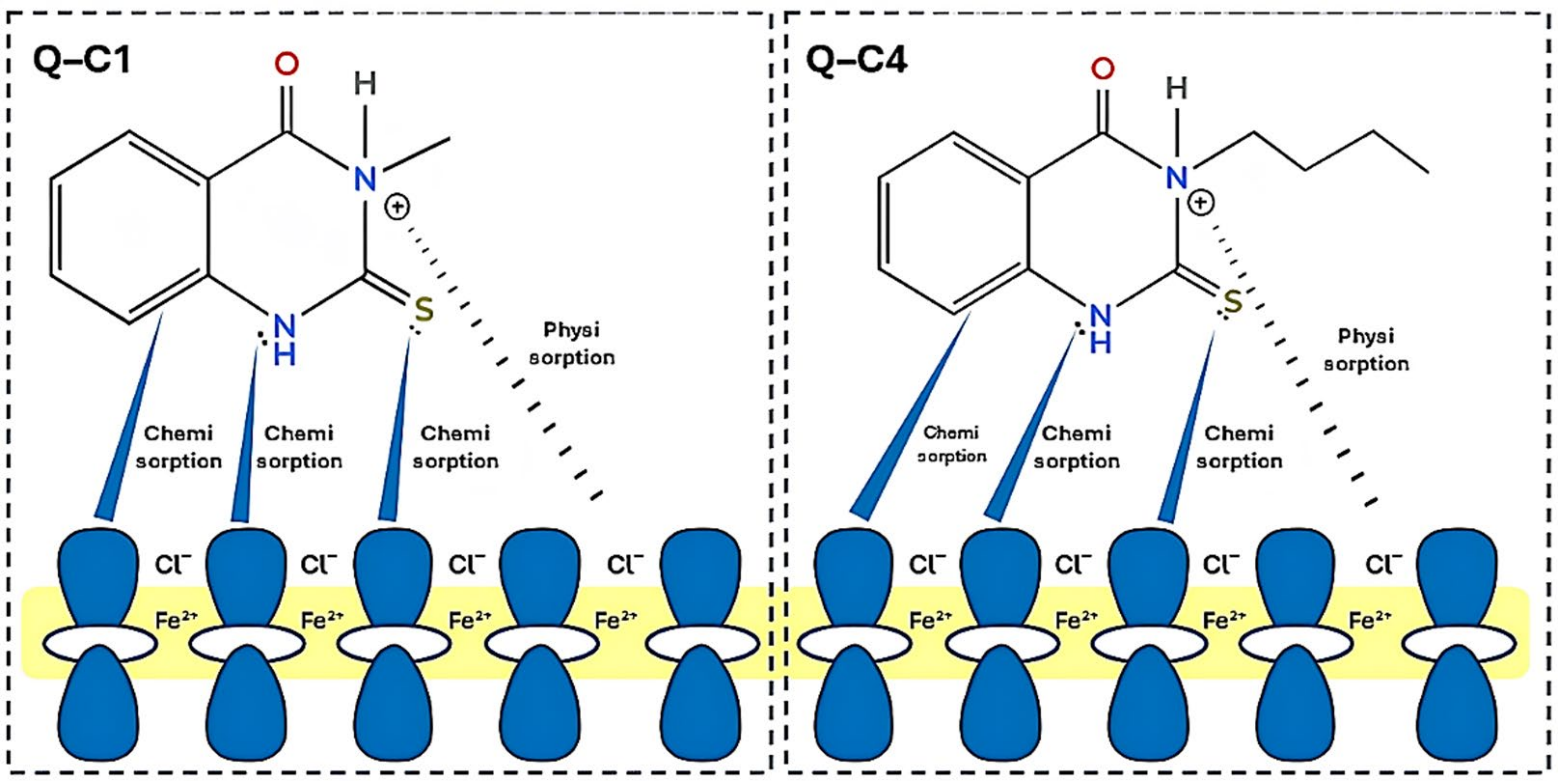
A schematic representation of the suggested mechanism of corrosion inhibition of quinazoline derivatives for CS in 1.0 M HCl.

The corrosion of metals and alloys typically involves two recognized processes at specific active sites: (1) anodic dissolution of the metal and (2) cathodic reactions, which include the development of hydrogen or the reduction of oxygen gases. Figure 18 illustrates a hypothetical mechanism of corrosion inhibition for the investigated quinazoline derivatives. The research outcome demonstrated that the inhibition mechanism includes the blocking of both locations on the CS surface by the inhibitor molecules, behaving as mixed-type inhibitors via an adsorption process. In the absence of inhibitors, the steel surface possesses a positive charge in acidic environments; thus, negative chloride anions attach to the surface of carbon steel. In the presence of organic molecules, the inhibitors’ adsorption onto the C-steel surface may arise via chemical or physical interactions. This is corroborated by the previously estimated adsorption Gibbs’ energy values.

Positively protonated organic compounds in the acidic solution and the negatively charged metal surface undergo an electrostatic interaction called physisorption. Additionally, the examined inhibitors can also be chemically adsorbed on the carbon steel surface in two mechanisms: The first way is achieved through the electron transfer of the unshared pairs of electrons in π-orbitals of heterocyclic rings to the vacant d-orbital of the iron atoms. The second way is accomplished via the chemical coordination between the heteroatoms of the inhibitor acting as electron donors and the iron atoms serving as electron acceptors, resulting in the formation of the Fe-inhibitor complex.

The degree of inhibition of the studied inhibitors is influenced by the length of the hydrophobic alkyl chain. An elongated alkyl chain will promote the buildup of a more compact protective layer on the CS surface, leading to increased surface coverage and enhanced corrosion mitigation.

## Conclusions

Our study emphasizes the synthesis of structurally simple quinazoline compounds with various alkyl substituents while incorporating key heteroatoms (N, S, and O) in a configuration favorable to adsorption and electron donation, thus offering cost-effective and scalable solutions for industrial protection from corrosion. The findings of this investigation can be clarified as follows:

1. The experimental and instrumental results indicate that the examined quinazoline compounds have good corrosion inhibition for C-steel in 1.0 M HCl.
2. The inhibitory efficiency of these derivatives rises with higher concentration and decreases with rising temperature, as shown by the ML method.
3. The adsorption of the inhibitor molecules on the carbon steel in 1.0 M HCl followed the Langmuir adsorption isotherm, resulting in the formation of a protective barrier over the surface, and the results further show the combination of both physisorption and chemisorption.
4. The PP data indicate a reduction in $\:{i}_{corr}$ values in solutions with Q-C1 and Q-C4 inhibitors relative to the uninhibited solution, alongside a shift to higher positive values of corrosion potential. Q-C1 and Q-C4 can be classified as mixed inhibitors.
5. The EIS study shows that the incorporation of Q-C1 and Q-C4 into the solutions results in a reduction in $\:{C}_{dl}$ and an elevation in $\:{R}_{ct}$ values relative to the blank solution, indicating the inhibitors’ adsorption on the metallic substrate.
6. The IE % of Q-C4 is higher than that of Q-C1 in 1.0 M HCl solution. This is attributed to the elongation of the alkyl substituent.
7. The surface analysis using SEM, FT-IR, and EDX confirmed the existence of a protective coating formed of adsorbed organic molecules, which protects CS from corrosive environments.
8. The theoretical calculations of quantum DFT reinforce the data derived from chemical and electrochemical methods.

## Supplementary Information

Below is the link to the electronic supplementary material.

Supplementary Material 1

## Data Availability

Raw data were generated at the Faculty of Science, Port-Said University, Egypt. Derived data supporting the findings of this study are available from the corresponding author, Prof. Dr. Samir A. Abd El-Maksoud, on request.
