# Supplementary material for: Effect of alkyl chain length on the corrosion inhibition performance of 2-thioxo-2,3-dihydroquinazolin-4(1H)-one derivatives for carbon steel in HCl solution
Source: Sci Rep. 2026 Mar 31;16:10982. doi: 10.1038/s41598-026-40197-z (PMC13043787; doi:10.1038/s41598-026-40197-z)

**The 1H and 13C NMR spectra of the synthesized compounds.**

**3-Methyl-2-thioxo-2,3-dihydroquinazolin-4(1*H*)-one (Fig.)** yellow crystals (1.8g, 92%), m.p 280^0^C. ^1^H-NMR spectrum, (400 MHz, DMSO-d6), δ, ppm (*J*, Hz): 12.93 (1H, bs, NH), 7.94-7.29 (4H, m, Ar-H), 3.64 (3H, s, NCH_3_). ^13^C-NMR (100.0 MHz, DMSO-d6), δ, ppm: 177.1 (CS), 161.4 (CO), 157.3, 147.2, 134.7, 125.8, 118.9 (C-Ar), 31.3 (NCH_3_). Found, %: C, 56.11; H, 4.05; N, 14.40; For C_9_H_8_N_2_OS (192.24). Calculated, %: C, 56.23; H, 4.19; N, 14.57.

**3-Butyl-2-thioxo-2,3-dihydroquinazolin-4(1*H*)-one (Fig.)** yellow crystals (1.99g, 86%), m.p 158^0^C. ^1^H-NMR spectrum, (400 MHz, DMSO-d6), δ, ppm (*J*, Hz): 12.90 (1H, bs, NH), 7.94-7.29 (4H, m, Ar-H), 4.38 (2H, t, *J* = 8.0 Hz, NCH_2_), 1.69-1.61 (2H, m, CH_2_), 1.38-1.29 (2H, m, CH_2_), 0.92 (2H, t, *J* = 8.0 Hz, CH_3_). ^13^C-NMR (100.0 MHz, DMSO-d6), δ, ppm: 176.6 (CS), 161.4 (CO), 157.8, 147.9, 134.6, 126.9, 125.6, 118.8 (C-Ar), 42.5 (NCH_2_), 29.4 (CH_2_), 20.4 (CH_2_), 13.8 (CH_3_). Found, %: C, 61.37; H, 5.91; N, 11.74 For C_12_H_14_N_2_OS (234.32). Calculated, %: C, 61.51; H, 6.02; N, 11.96

**
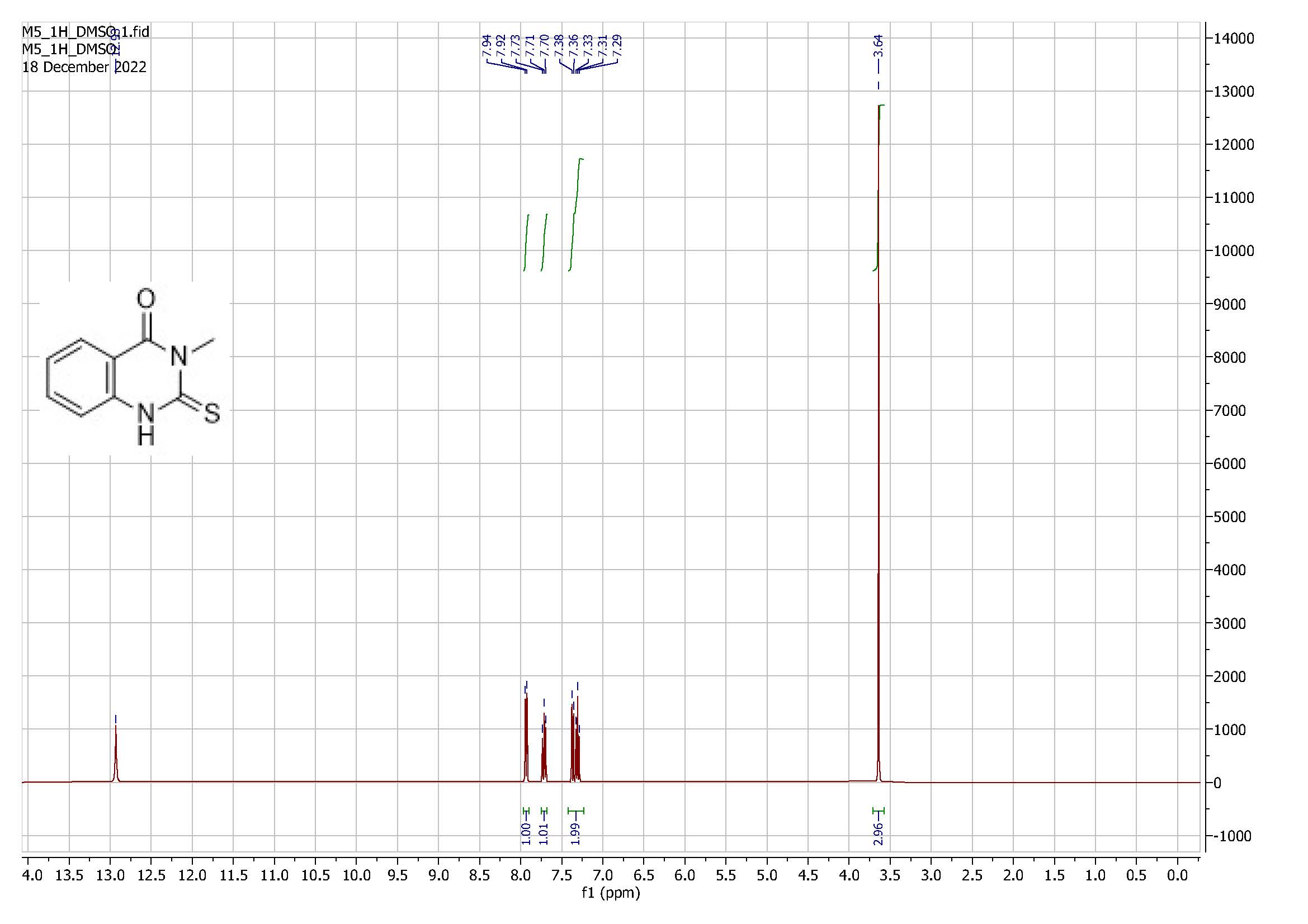
Figure S1:** H^1^ NMR spectrum of 3-methyl-2-thioxo-2,3-dihydroquinazolin-4(1*H*)-one.


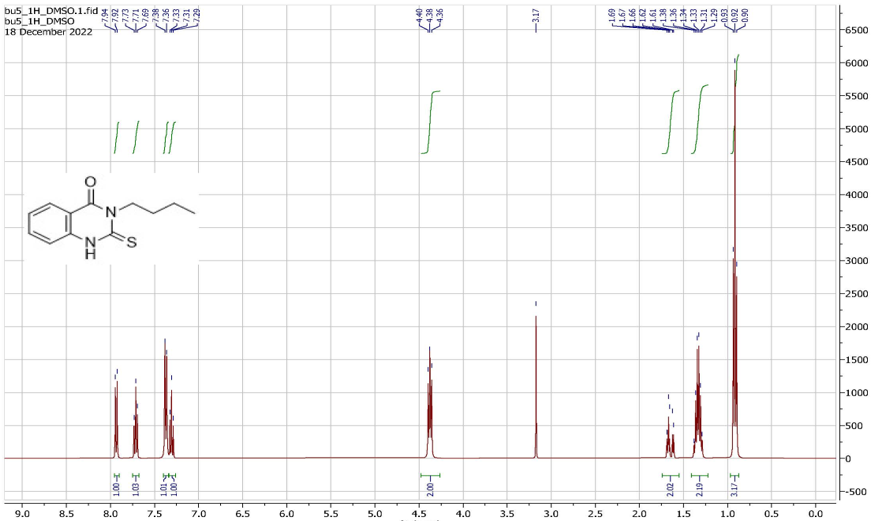


**Figure S2:** H^1^ NMR spectrum of 3-butyl-2-thioxo-2,3-dihydroquinazolin-4(1*H*)-one**.**

**Table S1.** Standard deviation values of corrosion rates of CS after 16 h in 1.0 M HCl at various temperatures, 298–318 K, and with different concentrations of inhibitors.

| **Inh.** | **Conc (M)** | **298 K** | | **303 K** | | **308 K** | | **313 K** | | **318 K** | |
| --- | --- | --- | --- | --- | --- | --- | --- | --- | --- | --- | --- |
|  |  | **CR**  **(mg cm ^-2^**  **min^-1^)** | $\boldsymbol{\pm}$**SD** $\boldsymbol{\times}$**10^3^** | **CR**  **(mg cm ^-2^**  **min^-1^)** | $\boldsymbol{\pm}$**SD** $\boldsymbol{\times}$**10^3^** | **CR**  **(mg cm ^-2^**  **min^-1^)** | $\boldsymbol{\pm}$**SD** $\boldsymbol{\times}$**10^3^** | **CR**  **(mg cm ^-2^**  **min^-1^)** | $\boldsymbol{\pm}$**SD** $\boldsymbol{\times}$**10^3^** | **CR**  **(mg cm ^-2^**  **min^-1^)** | $\boldsymbol{\pm}$**SD** $\boldsymbol{\times}$**10^3^** |
| Blank | 1.0 M HCl | 0.0066 | 0.30 | 0.0094 | 0.39 | 0.0133 | 0.19 | 0.0199 | 0.35 | 0.0297 | 1.96 |
| **Q-C1** | 1.0x10^-5^ | 0.0033 | 0.23 | 0.0051 | 0.13 | 0.0080 | 0.39 | 0.0120 | 0.17 | 0.0180 | 0.92 |
|  | 1.5x10^-5^ | 0.0028 | 0.38 | 0.0044 | 0.26 | 0.0069 | 0.41 | 0.0104 | 0.10 | 0.0157 | 0.54 |
|  | 2.0x10^-5^ | 0.0023 | 0.03 | 0.0037 | 0.10 | 0.0059 | 0.38 | 0.0091 | 0.12 | 0.0140 | 0.36 |
|  | 3.0x10^-5^ | 0.0016 | 0.07 | 0.0027 | 0.12 | 0.0046 | 0.20 | 0.0074 | 0.22 | 0.0120 | 0.92 |
|  | 4.0x10^-5^ | 0.0014 | 0.10 | 0.0023 | 0.08 | 0.0037 | 0.13 | 0.0059 | 0.32 | 0.0095 | 0.76 |
| **Q-C4** | 1.0x10^-5^ | 0.0011 | 0.06 | 0.0021 | 0.08 | 0.0037 | 0.20 | 0.0065 | 0.97 | 0.0112 | 0.31 |
|  | 1.5x10^-5^ | 0.0011 | 0.04 | 0.0019 | 0.04 | 0.0032 | 0.03 | 0.0051 | 0.52 | 0.0082 | 0.96 |
|  | 2.0x10^-5^ | 0.0009 | 0.03 | 0.0015 | 0.15 | 0.0024 | 0.31 | 0.0037 | 0.35 | 0.0057 | 0.41 |
|  | 3.0x10^-5^ | 0.0008 | 0.03 | 0.0013 | 0.12 | 0.0021 | 0.01 | 0.0034 | 0.49 | 0.0055 | 0.39 |
|  | 4.0x10^-5^ | 0.0007 | 0.07 | 0.0012 | 0.07 | 0.0020 | 0.17 | 0.0032 | 0.08 | 0.0052 | 0.20 |

**Figure S3:** A representative simulation of the Nyquist plot with the suggested model without an inhibitor.


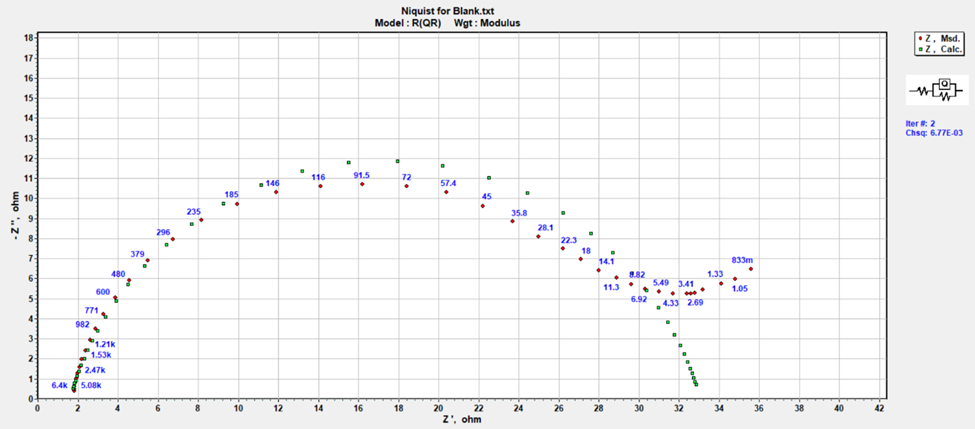

Supplement: Supplementary file 1 — Supplementary Material 1 [file 41598_2026_40197_MOESM1_ESM.docx]
